# Good manufacturing practice production of human corneal limbus-derived stromal stem cells and in vitro quality screening for therapeutic inhibition of corneal scarring

**DOI:** 10.1186/s13287-023-03626-8

**Published:** 2024-01-08

**Authors:** Mithun Santra, Moira L. Geary, Elizabeth Rubin, Michael Y. S. Hsu, Martha L. Funderburgh, Christine Chandran, Yiqin Du, Deepinder K. Dhaliwal, Vishal Jhanji, Gary Hin-Fai Yam

**Affiliations:** 1 Corneal Regeneration Lab, Department of Ophthalmology, University of Pittsburgh School of Medicine, Pittsburgh, PA USA; 2 Immunologic Monitoring and Cellular Products Laboratory, Hillman Cancer Centre, University of Pittsburgh School of Medicine, Pittsburgh, PA USA; 3 Department of Ophthalmology, University of South Florida, Tampa, FL USA; 4 McGowan Institute for Regenerative Medicine, University of Pittsburgh, Pittsburgh, PA USA; 5 Department of Ophthalmology, Mercy Vision Institute, University of Pittsburgh, 1622 Locust Street, Pittsburgh, PA 15219 USA

**Keywords:** Cornea scarring, Cell therapy, Corneal stromal stem cells, Good manufacturing practice, Quality control

## Abstract

**Background:**

Mesenchymal stem cells in the adult corneal stroma (named corneal stromal stem cells, CSSCs) inhibit corneal inflammation and scarring and restore corneal clarity in pre-clinical corneal injury models. This cell therapy could alleviate the heavy reliance on donor materials for corneal transplantation to treat corneal opacities. Herein, we established Good Manufacturing Practice (GMP) protocols for CSSC isolation, propagation, and cryostorage, and developed in vitro quality control (QC) metric for in vivo anti-scarring potency of CSSCs in treating corneal opacities.

**Methods:**

A total of 24 donor corneal rims with informed consent were used—18 were processed for the GMP optimization of CSSC culture and QC assay development, while CSSCs from the remaining 6 were raised under GMP-optimized conditions and used for QC validation. The cell viability, growth, substrate adhesion, stem cell phenotypes, and differentiation into stromal keratocytes were assayed by monitoring the electric impedance changes using xCELLigence real-time cell analyzer, quantitative PCR, and immunofluorescence. CSSC’s conditioned media were tested for the anti-inflammatory activity using an osteoclastogenesis assay with mouse macrophage RAW264.7 cells. In vivo scar inhibitory outcomes were verified using a mouse model of anterior stromal injury caused by mechanical ablation using an Algerbrush burring.

**Results:**

By comparatively assessing various GMP-compliant reagents with the corresponding non-GMP research-grade chemicals used in the laboratory-based protocols, we finalized GMP protocols covering donor limbal stromal tissue processing, enzymatic digestion, primary CSSC culture, and cryopreservation. In establishing the in vitro QC metric, two parameters—stemness stability of ABCG2 and nestin and anti-inflammatory ability (rate of inflammation)—were factored into a novel formula to calculate a Scarring Index (SI) for each CSSC batch. Correlating with the in vivo scar inhibitory outcomes, the CSSC batches with SI < 10 had a predicted 50% scar reduction potency, whereas cells with SI > 10 were ineffective to inhibit scarring.

**Conclusions:**

We established a full GMP-compliant protocol for donor CSSC cultivation, which is essential toward clinical-grade cell manufacturing. A novel in vitro QC–in vivo potency correlation was developed to predict the anti-scarring efficacy of donor CSSCs in treating corneal opacities. This method is applicable to other cell-based therapies and pharmacological treatments.

**Supplementary Information:**

The online version contains supplementary material available at 10.1186/s13287-023-03626-8.

## Introduction

Corneal blindness is a leading cause of vision loss worldwide. Using the definition of blindness by World Health Organization, over 240 million people worldwide suffer from moderate-to-severe vision impairment due to corneal opacities, and about 10 million individuals have corneal blindness (https://www.who.int/news-room/fact-sheets/detail/blindness-and-visual-impairment). In some locations of Africa, nearly 90% of total blindness cases are related to corneal pathology [1]. Corneal opacification or scarring occurs after trauma, corneal ulcers, infections, surgeries or secondary to other corneal diseases or disorders, including congenital corneal dystrophies and degenerations (e.g., keratoconus and ectasia). At present, safe and reliable treatments are limited. Topical corticosteroids and agents, such as mitomycin C, are used to treat or prevent corneal haze; however, there are variable efficacy and known adverse effects of these treatments. In addition, the safety, dosing and duration of these treatments are subjects of debate among clinicians. Corneal transplantation (penetrating and deep anterior lamellar keratoplasties) using donor corneas remains the most popular choice for replacement of scarred corneas in order to restore corneal functions and vision. About 185,000 corneal transplants are performed annually in 116 countries [2]. The limited global supply of transplantable donor corneas, immune response, risks of long-term graft rejection, and potential surgical complications however restrict the widespread use of keratoplasty. With an estimate of 12.7 million people waiting for cornea transplantation worldwide, only one in 70 of the needs has access to donor tissues [2]. In recent years, cell-based therapy has shown promise as a potential treatment option for patients with mild-to-moderate corneal opacities. In a phase 1 clinical trial, the intrastromal injection of autologous adipose-derived mesenchymal stem cells (Ad-MSCs) or implantation of Ad-MSC-populated stromal laminae proved to be safe. The treatment demonstrated stabilization of corneas affected by advanced keratoconus and even with new collagen production [3, 4]. Mid-term outcome at 3-year post-treatment revealed no ocular complications and an improved uncorrected visual acuity and reduced corneal densitometry [5]. Currently, as per the clinicaltrials.gov, four clinical trials for cell-based therapy in corneal opacity are undergoing at different sites; three in LV Prasad Eye Institute, Hyderabad, India, and one in Massachusetts Eye and Ear Infirmary, Boston, USA.

Previous pre-clinical studies using mouse models with acute stromal injury created by mechanical debridement, or burns caused by alkali or liquid nitrogen, have demonstrated the therapeutic potential of human corneal stromal stem cells (CSSCs) in inhibiting corneal scarring [6–9]. The administration of cells, topically with fibrin gel or via intrastromal injection, to wounded corneas suppressed fibrosis (shown by the downregulation of repair-type collagen III [Col3], fibronectin [FN], tenascin C [TNC], and α-smooth muscle actin [αSMA]), and reduced opacity-related light scattering, resulting in decreased haze formation and improved corneal clarity. Inside the corneal stroma, the transplanted CSSCs restored the uniformity of collagen lamellae with a regular pattern of collagen fibrils [6, 9]. Several mechanisms of CSSC’s anti-scarring effects have been discerned. As keratocyte progenitors, CSSCs differentiate and express keratocyte-specific collagens and proteoglycans (keratocan, lumican, and decorin) that comprise the stromal matrix [6, 10]. They also produce matrix metalloproteinases (MMP), which break down the excess collagen linking in scar tissues [11]. Moreover, the treatment upregulated tumor necrosis factor α stimulated gene 6 (TSG-6) inhibiting CD11b + /Ly6G + neutrophil infiltration, hence suppressing stromal fibrosis [12]. The production of TGFβ3 by CSSCs further reduced the expression of inflammatory genes (CD80, CXCL5, and PAUR) [13]. Additionally, CSSC secretome, when applied to mouse corneal wounds, suppressed inflammation (reduced CD45 + and CD11b + neutrophils), downregulated fibrosis genes (Col3a1, SPARC, αSMA) and was neuroprotective [14]. Examination of the purified fraction of extracellular vesicles (EV) produced by human CSSCs revealed a cargo of microRNAs. Depleting global miRNA content in EV using specific siRNA-mediated Alix knockdown to block endosomal sorting complex required for transport ESCRT, CSSCs lost the scar-reducing effects in vivo [15]. Recently, two anti-fibrotic miRNAs (miR-29a and 381) were identified in CSSC-derived EV, and in vivo treatment with cells overexpressing both miRNAs reduced corneal fibrosis (downregulated Col3A1, FN, and αSMA), resulting in an improved corneal clarity [16].

Based on these encouraging outcomes from pre-clinical studies, CSSC therapy holds promise as a potential strategy for addressing corneal opacities and scarring. However, not every batch of donor CSSCs, following ex vivo expansion, exhibits suitable anti-scarring and healing activity. Therefore, it is necessary to identify variations in CSSC effectiveness and select the suitable batch of cells for potential clinical use. In our culture protocol, the primary donor CSSCs are propagated at passage 0 (P0) and P1 to obtain seed stocks, which are then used to produce large quantities of distribution stocks at P2 and P3. The P2 cells are characterized for CSSC enrichment and are utilized for quality control (QC) assays to determine the suitability of P3 cells for treatment purposes. Previous reports have indicated that donor CSSCs expanded beyond P3 experienced a gradual loss of therapeutic potential [6]. As a result, our work focused on evaluating P2 CSSCs for relevant in vitro cellular indicators and establish QC metrics to correlate ex vivo expanded CSSC products with their in vivo healing potency and anti-scarring effects. These QC measures can be used to further establish CSSC product release criteria when cells are produced under current good manufacturing practices (cGMP).

## Methods

### Study design

This study had two key objectives. The first one was to demonstrate the robustness of refined CSSC isolation and culture under a cGMP environment as this would establish the readiness of cGMP production of CSSCs for an FDA investigational new drug (IND)-based clinical trials. The second objective involved identifying functionally correlating biomarkers for ex vivo expanded CSSC that demonstrate good in vivo healing and anti-scarring potency. The functionality of qualified GMP-raised CSSCs (CSSC_[GMP]_) was demonstrated using a pre-clinical mouse model of anterior stromal injury, thus highlighting its practicability in clinical applications for treating corneal scarring.

### Human corneoscleral tissues and donor selection criteria

Research-grade corneal tissues from de-identified donors obtained through the Center for Organ Recovery and Education (CORE), Pittsburgh, PA, and EverSight Eye Bank were procured with informed consent from the next of kin of all deceased donors regarding tissue donation for research. Donors included in this study had no history of ocular diseases, cancer or drug use, and tested negative for transmissible diseases (including causes by human immunodeficiency virus, hepatitis virus B and C, and syphilis). The corneas were free from any known diseases, injuries, or inflammation. Enucleation and cornea harvest procedures were performed by trained technicians under sterile conditions from eye banks. The time from donor death to corneal preservation in Optisol-GS (Bausch & Lomb, Rochester, NY) was less than 12 h, and the corneas were promptly processed upon arrival at the culture facility. A total of 24 donor corneal tissues at 42.3 ± 18.6 years old were used in this study (Additional file 1: Table S1). They had corneal endothelial cell count greater than 2,000 cells per mm^2^.

### Human CSSC isolation and primary culture with a research-based protocol

The anterior limbal stroma (0.5 to 1 mm width, 10–20 µm depth) free from epithelium was isolated under stereomicroscopy. The thin tissue was cut into six to eight pieces and digested with DMEM/F12 (Thermo Fisher Sci, Waltham, MA) containing collagenase A (1 mg/ml, Roche, Indianapolis, IN) for 6 to 8 h at 37 °C with gentle rotation at 30 rpm, following our published protocol (Table 1) [6, 9]. The resulting digest was filtered through a 40-µm cell strainer, and then centrifuged at 300 g for 5 min. After several washes in basal medium, primary cells were suspended in stem cell growth medium (JM-H) (Table 2) and seeded onto a culture surface pre-coated with FNC mix (Athena Enzyme Systems, Baltimore, MD). Cells at passage 0 (P0) exhibiting clonal growth were collected following treatment by TrypLE Select (Thermo Fisher). Subsequently, 250 cells were seeded in a 75 cm^2^ FNC-coated culture flask (~ 5 cells/cm^2^).

**Table 1:** Comparison of qualified GMP-grade manufacturing reagents to research-grade reagents for key culture steps

	Research protocol	GMP protocol
Limbal tissue digestion	Collagenase A (1 mg/ml; Roche #11,088,793,001)	Collagenase I (Nordmark NB6 GMP, #N0002779)
Culture surface coating	FNC mix (Athena Enzyme #0407)	Recombinant human fibronectin (R&D #4305B-GMP)
Cell detachment	TrypLE Select (Gibco #12,563,011)	TrypLE Select CTS (Gibco #A12859)
Cryopreservation	Dimethyl sulfoxide (DMSO; 5%; Sigma #D2650)	CryoStor®® DMSO (5%; Biolife Solutions Ltd. #210,373)

**Table 2:** Culture medium formulation (research versus GMP-grade)

Research-based JM-H medium	Qualified GMP reagents
DMEM, 1 g/L L-glucose (Gibco #10,567–014): MCDB201 (Sigma-Aldrich #M6770) mixed in a 3:2 (vol/vol) ratio	DMEM, 1 g/L L-glucose (Gibco #10,567–014): Ham F12 (Gibco #31,765–035) mixed in a 3:2 (vol/vol) ratio
Antibiotic–antimycotic (1%; Gibco #14,140–122)	Antibiotic–antimycotic (1%; BioWhittaker, Lonza #17-745E)
AlbuMAX I (0.1%; Gibco #11,020–021)	Flexbumin (0.1%; BioSupply #00944–0493-01)
L-ascorbate-2-phosphate (0.5 mM; Sigma-Aldrich #A8960)	L-ascorbic acid (0.5 mM; Tocris Biosci #5778)
Recombinant human EGF (10 ng/mL; Sigma-Aldrich #E9644)	Recombinant human EGF (10 ng/mL; Gibco #PHG6045)
Recombinant human PDGF-BB (10 ng/mL R&D #520-BB)	Recombinant human PDGF (10 ng/mL; R&D #220-GMP-010)
Dexamethasone (10 nM; Sigma-Aldrich #D4902)	Dexamethasone (10 nM; micronized USP, Spectrum #DEE121)
Human serum (2%; Innovative Res #ISER-36670)	Human serum (2%; Innovative Res #ISER-36670)

Primary human stromal keratocytes were isolated from central stroma obtained from a total of five donors, Additional file 1: Table S1). The tissue was digested with 0.1% collagenase A for 6 h at 37 °C. Single cell suspension was obtained by filtering the digest through a 40 µm cell strainer. The isolated keratocytes were then expanded using our reported protocols [17–19]. Once the cells reached passage 3–4, the cultures were placed in serum-free condition for 7 to 10 days to generate *bona fide* keratocytes. On the other hand, stromal fibroblasts (SF) were derived from keratocytes cultured with DMEM/F12 plus 10% fetal bovine serum (FBS, Gibco) for 2 passages.

### Cell viability assay after tissue digestion by collagenase

Anterior limbal stromal tissues obtained from six donor corneas were trimmed to tiny pieces, pooled together, and then separated into three portions. Each portion was digested using a different collagenase type (1 mg/ml): (i) collagenase A (research-grade, Roche #11,088,793,001), (ii) Celase (GMP, Worthington #1235–01), or (iii) NB6 (GMP, Nordmark #N0002779), at 37 °C for 6 h (Table 1). After the digest was filtered through a 40-μm cell strainer, followed by washes and centrifugations, the resulting cell pellet was resuspended in sterile PBS (2 ml volume), loaded to a Ficoll-Paque gradient (4 ml volume), and centrifuged at 1,000 g for 20 min. This step removed tissue debris, and the cell enriched fraction in the interface layer was harvested. After PBS washes, the cells were resuspended in JM-H medium (0.5 ml). Viability count was performed by 0.4% trypan blue dye exclusion using a Countess Automatic Cell Counter (Invitrogen). Each sample was counted three times, and the mean ± standard deviation (SD) was calculated.

### Cell adhesion and growth assays

The adherence of CSSCs to culture surfaces with different coatings was evaluated using xCelligence real-time cell analyzer RTCA SP (Agilent/ACEA, Santa Clara, CA). CSSCs at P2 were seeded at a density of 4 × 10^3^ cells in each well of an E-plate 96, which was pre-coated with one of the following (i) FNC (research-grade, as positive control), (ii) recombinant human fibronectin (hFn, 50 µg/ml; R&D #3420–001-03), (iii) recombinant human laminin-521 (hLn-521, 50 µg/ml; Thermo Fisher #A29249), or (iv) uncoated (as negative control) (Table 1). The experiments were conducted in triplicate, and the impedance value of each well was automatically recorded every 15 min for a period of 32 h and expressed as a Cell Index (CI) value. The rates of adhesion at 8 and 24 h were determined by comparing the mean normalized CI values among groups.

### Clonal expansion and colony-forming efficiency

CSSCs were seeded at 5 cells/cm^2^ in a tissue culture plate (100-mm diameter) pre-coated with hFn, i.e., ~ 400 cells per dish. Fresh medium was replenished every 3–4 days. At day 10, cells were fixed with ice-cold methanol for 10 min. After PBS washes, the cells were stained with 0.1% crystal violet in 20% ethanol for 5 min. After rinses with distilled water and brief drying, the entire plate was photographed and clones with > 50 cells were quantified. The colony-forming efficiency was calculated by the number of clones over the number of cells seeded.

### Optimization of GMP reagents in CSSC culture

To identify the cellular safety of GMP alternatives in CSSC culture, our study incorporated three established CSSC cultures at P2. Initially, the cultures were screened in JM-H medium where one component was substituted with the corresponding GMP reagent (e.g., rhEGF from Sigma #E9644 to GMP-grade rhEGF from Gibco #PHG6045). The cell growth was assessed using xCELLigence RTCA for up to 120 h. We determined the suitability of GMP reagents for CSSC culture by comparing their minimal impedance changes compared with the JM-H control protocol, which served as an indicator of their safety and compatibility with CSSC features and overall cellular health status (Table 2).

### Cryopreservation and cell survival, adhesion and growth assays

To assess the suitability of GMP- grade cryopreservation media, donor CSSCs at P1 from three corneas were utilized. Four different freezing media were evaluated: (i) Stem-CellBanker (SCB, DMSO-free & xeno-free; Amsbio #13,925); (ii) CryoStor® CS10 (serum-free with 10% DMSO; Biolife Solutions Ltd. #210,373); (iii) Cryopres™ USP DMSO (CP, MP Biomed. #27,801–45), in comparison with the (iv) conventional freezing medium made up of 5 to 10% DMSO (Sigma) in JM-H medium. CSSCs at a density 2 × 10^5^ cells were resuspended in each of these freezing media after dissociation with TripLE Select and washes. The cell suspensions were placed in labeled cryovials. The freezing process for cells in CryoStor® CS10, and the same diluted to 5% with JM-H (CS5), Cryopres™ DMSO (5% and 10% prepared with JM-H) (CP10 and CP5) were conducted with controlled freezing using a freezing container (Nalgene) with isopropyl alcohol (Merck Millipore, Billerica, MA). The cells in cryovials were left at -80 °C for 24 h, then transferred to liquid N_2_ (vapor-phase) for up to 12 months. In contrast, the cells suspended in DMSO-free SCB were directly frozen at -80 °C, and then transferred to N_2_ vapor-phase. At defined time intervals, cryovials were retrieved and thawed at 37 °C. Following dilution with JM-H medium (9 volumes) and centrifugation, the cell pellets were resuspended in JM-H for various assays.

1. Cell attachment: seeding 20 cells per mm^2^ on hFn-coated culture surface. After 24 h, the attached cells were quantified in 5 random microscopic fields and the percentage of cell attachment with reference to 315 cells per field with 2048 × 1536 pixels in dimension were calculated.
2. Seeding at 2 × 10^3^ cells per well of an E-plate 96 pre-coated with FNC to monitor cell growth by examining the electrical impedance changes up to 120 h.
3. Remaining cells were stained with a Fluorescein isothiocyanate (FITC) Annexin V apoptosis detection kit with propidium iodide (PI) (BioLegend, San Diego, CA) following manufacturer’s instructions. The stained cells were analyzed with flow cytometry using Cytoflex LX (Beckman Coulter) and data analysis by Floreada.io. The percentages of live cells (Annexin V and PI negative) were compared among different cryopreservation.

### In vitro differentiation of CSSCs to stromal keratocytes

Human CSSCs at P3 were seeded at a density of 2 × 10^3^ cells/cm^2^ on Col I-coated culture surface. After allowing to attach for 24 h, the cells were differentiated to keratocytes by incubating with DMEM containing GlutaMAX I, 1 g/L D-glucose and sodium pyruvate (Thermo Fisher), ascorbate-2-phosphate (1 mM; Sigma), bFGF (10 ng/ml, Gibco), and transforming growth factor β3 (TGFβ3, 0.1 ng/ml; Gibco) [20], and replenished with fresh medium every 2 to 3 days. At day 7, the cells were assayed for keratocyte gene expression, including immunostaining of keratocan with rabbit anti-human Kera (Sigma #HPA039331), and qPCR for AQP1, B3GnT7, CHST6, and lumican (Lum) (primer information in Additional file 1: Table S2) [9].

### Immunofluorescence

Cells were fixed with 2% freshly prepared neutral-buffered paraformaldehyde for 10–15 min at room temperature (RT), followed by PBS washes. After quenched with ice-cold 50 mM ammonium chloride (Sigma-Aldrich) for 5 min on ice and PBS washes, cells were treated with endo-β-galactosidase (Sigma; 0.5 U/ml PBS) for 20 min at 37 °C to remove keratan sulfate side chains. Samples were then permeabilized and blocked with 0.15% saponin (Sigma), 2% bovine serum albumin (BSA, Sigma), and 5% normal goat serum (Gibco) in PBS for 30 min, followed by incubation with mouse-anti-human monoclonal antibody against AQP1 (1 µg/ml; Santa Cruz Biotech, CA) and rabbit anti-human antibody against keratocan (Kera, 1:150 dilution; Millipore) for an hour at RT. After PBS rinses, signals were detected by species-specific Alexa Fluor™ 488 and 594-conjugated IgG antibodies (Jackson Immunoresearch Lab, West Grove, PA) for an hour in dark. The rinsed samples were mounted in FluoroShield containing 4,6-diamidino-2-phenylindole (DAPI) and examined under fluorescence microscopy (IX83, Olympus).

### Chronic pro-inflammatory macrophage-osteoclastogenesis assay

CSSCs at P2 at ~ 50% confluence were cultured for 4 days with serum-depleted JM (Additional file 1: Fig. S1). The conditioned media (CM) was spun in MicroCon filter (YM-10 membrane, Millipore) at 4000 g at 4 °C until a final volume about 1/20th of the original volume (CM concentrate, CMconc). The total protein content was determined using a Pierce BCA Protein Assay kit (Thermo Fisher). Half of the CMconc was heat-denatured at 95 °C for 5 min while the remaining portion was kept as native. Mouse macrophages RAW264.7 (American Type Cell Collection, Manassas, VA) were plated at 10^5^ cells/cm^2^ in DMEM/F12 with 10% heat-inactivated fetal bovine serum (FBS, Gibco). At ~ 50% confluence, RAW cells were treated with RANK ligand (RANKL) peptide (50 ng/ml, Sigma) and concanavalin A (conA, 20 µg/ml, Sigma), in the presence of native or heat-denatured CMconc (500 µg protein). After day 5, the expression of mouse tartrate-resistant alkaline phosphatase (ACP5), cathepsin K (CTSK) and matrix metalloproteinase 9 (MMP9) genes (Additional file 1: Table S2) in RAW cells was examined. After triplicate runs, the relative RNA abundance was determined using the 2^−ΔΔCT^ method after normalization with 18S, and the fold changes between treatments with native versus denatured CMconc samples were obtained. The rate of inflammation (RInflam) was calculated as the sum of the fold change ratio (native versus denatured) of all three genes.

RInflam = ACP5(naïve/denatured) + MMP9(naïve/denatured) + CTSK(naïve/denatured).

### Mouse corneal injury model and CSSC treatment

A total of 168 BALB/c mice (breeders from Charles River Lab, Wayne, PA), aged 8 to 10 weeks old, were used. They were anesthetized with intraperitoneal ketamine (50 mg/kg) and xylazine (5 mg/kg) [8]. The right eyes received topical proparacaine hydrochloride (0.5%, Alcaine®, Alcon, Fort Worth, TX) for local analgesia. All procedures and cell treatments were performed by GY. In brief, the central 2-mm corneal epithelium was removed using a high-speed rotation with an AlgerBrush II (Accutome Inc, Malvern, PA, US). The basement membrane and anterior stroma of 10 to 20 µm depth was ablated by a second burring (Additional file 1: Fig. S2). After cleaning, the wound surface was overlaid with 0.5 µl fibrinogen (37 mg/ml, Sigma-Aldrich) containing 5 × 10^4^ CSSCs, followed by 0.3 µl thrombin (100 U/ml, Sigma-Aldrich) [9]. Each CSSC batch was used for eight corneas, resulting in a total of 144 corneas were treated with CSSC, while the control groups consisted of fibrin-only and non-treated wound corneas. The treated eyes received topical tobramycin ophthalmic solution (0.3%, USP, Somerset Therapeutics, Hollywood, FL) 3 times daily for 3 days.

### Ophthalmic examination and assessment of corneal scarring

Before and after injury, as well as at weekly intervals, a cross-sectional corneal scanning (4 × 4 mm area) was performed using a Spectral Domain Optical Coherence Tomography (SD-OCT, Envisu R2210, Leica [Bioptigen], Morrisville, NC). The obtained images were processed with NIS-Elements software (Nikon, Melville, NY) in a masked fashion. Corneal thickness was measured by taking the average of three measurements obtained at the center (0 mm) and at 0.5 mm on both sides [18]. On day 10, the mice was euthanized by administering an overdose of intraperitoneal sodium pentobarbital injection, followed by cervical dislocation. All isolated corneas were imaged, and the scar area was determined using Fiji, an open-source image analysis software package (https://fiji.sc/), in a blinded manner [8].

### Gene expression

Cells were collected in RLT buffer (Qiagen) with freshly added β-mercaptoethanol (β-ME; Sigma). Total RNA was extracted using RNeasy Miniprep kit (Qiagen) with on-column RNase-free DNase kit (Qiagen), following manufacturers’ instructions. After quantification by Nanodrop (Thermo Fisher), total RNA (500 ng) was reverse-transcribed with SuperScript III Reverse Transcriptase kit (Thermo Fisher) and random primer hexanucleotides (10 ng/ml, Thermo Fisher). Complementary DNA (cDNA) was assayed for gene expression using SYBR Green Real-Time Master Mix (Life Technol, Carlsbad, CA) and specific primer (Additional file 1: Table S2) in a QuantStudio 3 real-time PCR system (Applied Biosystems, Thermo Fisher). The experiments were run in triplicate. The relative RNA abundance was determined by ΔCT method after normalization with the housekeeping 18S. A similar protocol was followed for mouse corneal samples with an additional step of mechanical tissue disruption using MagNA Lyser beads in a MagNA Lyser Instrument (Roche). The expression fold changes were presented as mean ± SD. Significance was determined by the nonparametric Mann–Whitney U test.

### Statistics

All experiments were performed in triplicate, and each group had eight or more animals, determined based on sample size calculation using the formula n = 1 + 2C(s/d)^2^, where s is the standard deviation (SD), d is the difference to be detected, and C is the constant dependent on the value of α (significance P level) and β (1-power) [21]. The constant C is 10.51 with P at 0.05 (α) and power (1 − β) at 0.9. Assuming an effect size and SD are set to 30% and 20%, respectively, the calculated sample size is n = 8 per group. The data were presented as mean ± SD. Mean values were compared using unpaired two-tailed Student’s t test or ANOVA with a post hoc Bonferroni test using GraphPad Prism 7 software. Nonparametric comparisons were done by Mann–Whitney U test.* P* < 0.05 was considered statistically significant.

## Results

### Isolation of anterior limbal stroma and collagenase digestion

**Fig. 1:**
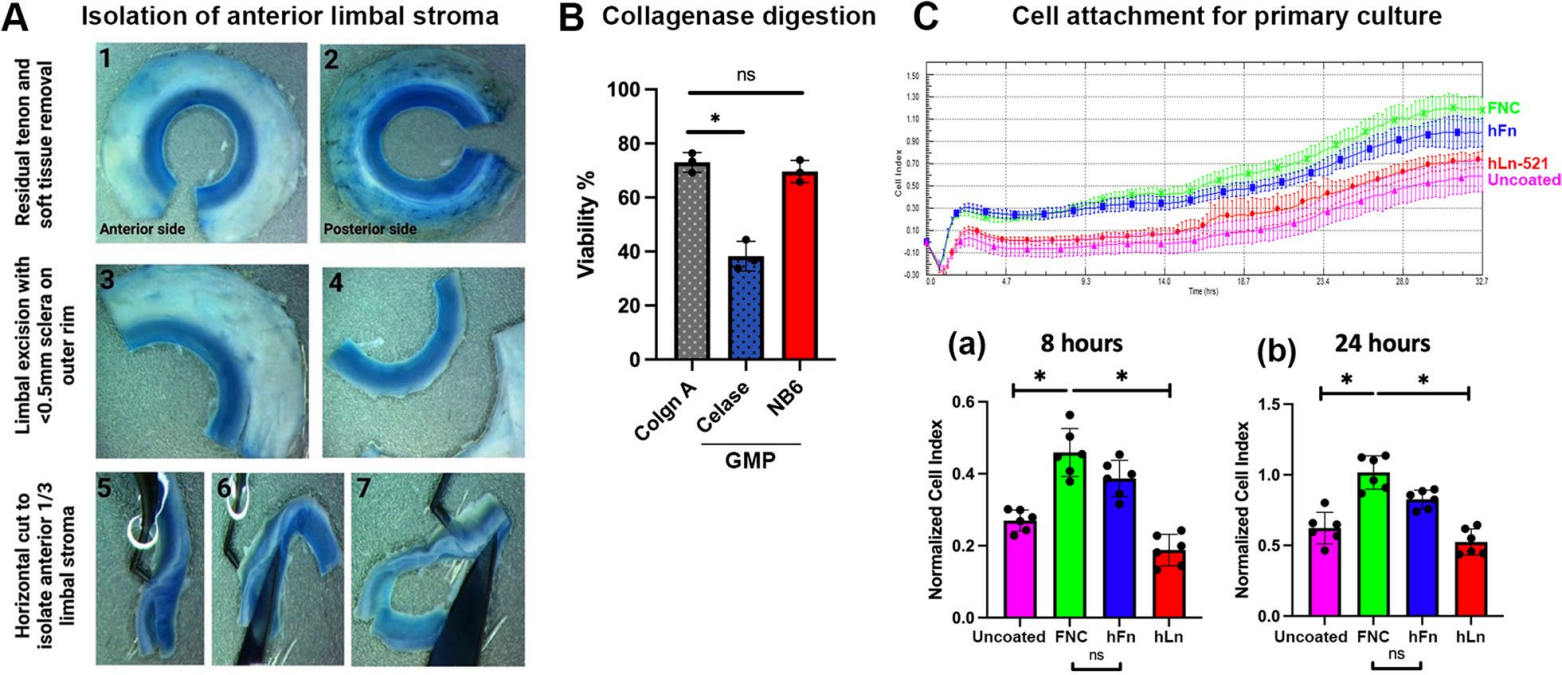
**A** Steps of donor corneoscleral limbal tissue processing and isolation of anterior limbal stroma. **B** Tissue digestion—cell viability by trypan blue dye exclusion assay for freshly isolated cells after the pooled limbal stromal tissue digested with collagenase A (Collgn A, research-grade) from Roche, and NB6 (GMP collagenase I from Nordmark) were insignificantly different (ns). A significant drop in cell viability was observed with Celase (GMP collagenase from Worthington) digestion (*P < 0.01, One-way ANOVA). **C** Cell attachment profile by xCELLigence revealed similar efficiency between FNC and hFn coating. Normalized impedance readings were extracted at (a) 8 and (b) 24 h. Similar attachment efficiencies of CSSCs were found on FNC and hFn pre-coated surface (ns—not significant difference). CSSC attachment was suboptimal on hLn-521 and non-coated surface (*P < 0.05, One-way ANOVA)

Following the step-by-step cleaning of non-stromal tissues, such as corneal and limbal epithelium, endothelium, iris and posterior limbus, the limbal rim with about 0.5 mm on both sides was isolated and cut into halves (Fig. 1A). The thin tissue strip was placed horizontally and trimmed to obtain the anterior 1/3 limbal stroma. After multiple PBS washes, each quarter was cut into about 1-mm^3^ pieces for collagenase digestion.

Our initial evaluation has optimized the enzyme working condition—collagenase A (Roche #11,088,793,001) at a concentration of 1 mg/mL for 6 to 8 h at 37 °C to digest the anterior limbal stroma and release CSSCs [6, 9]. We used this condition to compare with GMP-grade collagenases and assayed the cellular viability (Table 1). The digests were filtered and debris was removed through Ficoll-Paque gradient centrifugation. Cells at the interface layer were collected and the viability was assayed with trypan blue dye exclusion. Our results showed that tissues digested with GMP-grade NB6 collagenase (Nordmark) yielded viable CSSC (69.6 ± 4.1%), at a similar level as collagenase A (Roche) (72.1 ± 3.5%) (Fig. 1B). However, digestion with Celase (Worthington) resulted in significantly lower viability (38.3 ± 5.5%) (*P* < 0.05, one-way ANOVA).

### CSSC attachment with extracellular matrices

We compared the effectiveness of two GMP-grade ECM coatings, hFn (R&D #4305B-GMP) and hLn-521 (Thermo Fisher #A29249), with the laboratory-based protocol that used FNC mix coated on the culture surface to enhance CSSC attachment, which was evaluated by xCELLigence scans for up to 32 h (Fig. 1C). The attachment profiles at 8 and 24 h revealed that hFn coating yielded a similar efficiency in CSSC attachment as FNC coating. In contrast, the adherence of cells to the hLn-521 coated surface was significantly reduced. Uncoated surface (blank control) exhibited lower adhesion efficiency for CSSC.

### In-process CSSC propagation with GMP-grade medium formulation

Our primary goal in optimization the GMP protocol was to show the robustness of culture refinement and ensure compliance with regulatory standards in order to generate *bona fide* clinical-grade CSSCs. Over the past decade, various in vitro assessments, including clonal growth, stemness characteristics and differentiation to stromal keratocytes, have optimized the JM-H medium with defined composition to expand donor CSSCs [6, 10, 13, 22, 23]. The propagated cells were validated to exhibit in vivo scar inhibitory effects using animal models of corneal injury [6, 8, 9, 12, 24]. Therefore, we sought to identify suitable GMP alternatives for each JM-H component by assessing their effects on CSSC stability and growth. This testing ensures that the qualified CSSC product under GMP-aligned preparation—CSSC_(GMP)_, contains the correct cellular features as previously described. Substituting JM-H with other commercially available xeno-free and serum-free stem cell culture media may save time and effort, its different composition however carries the risk of generating cells with altered characteristics. This deviation from the established cellular features poses significant risks for future clinical applications. Therefore, our aim was to maintain the consistency and reliability of CSSC products throughout the transition to GMP-compliant protocols.

**Fig. 2:**
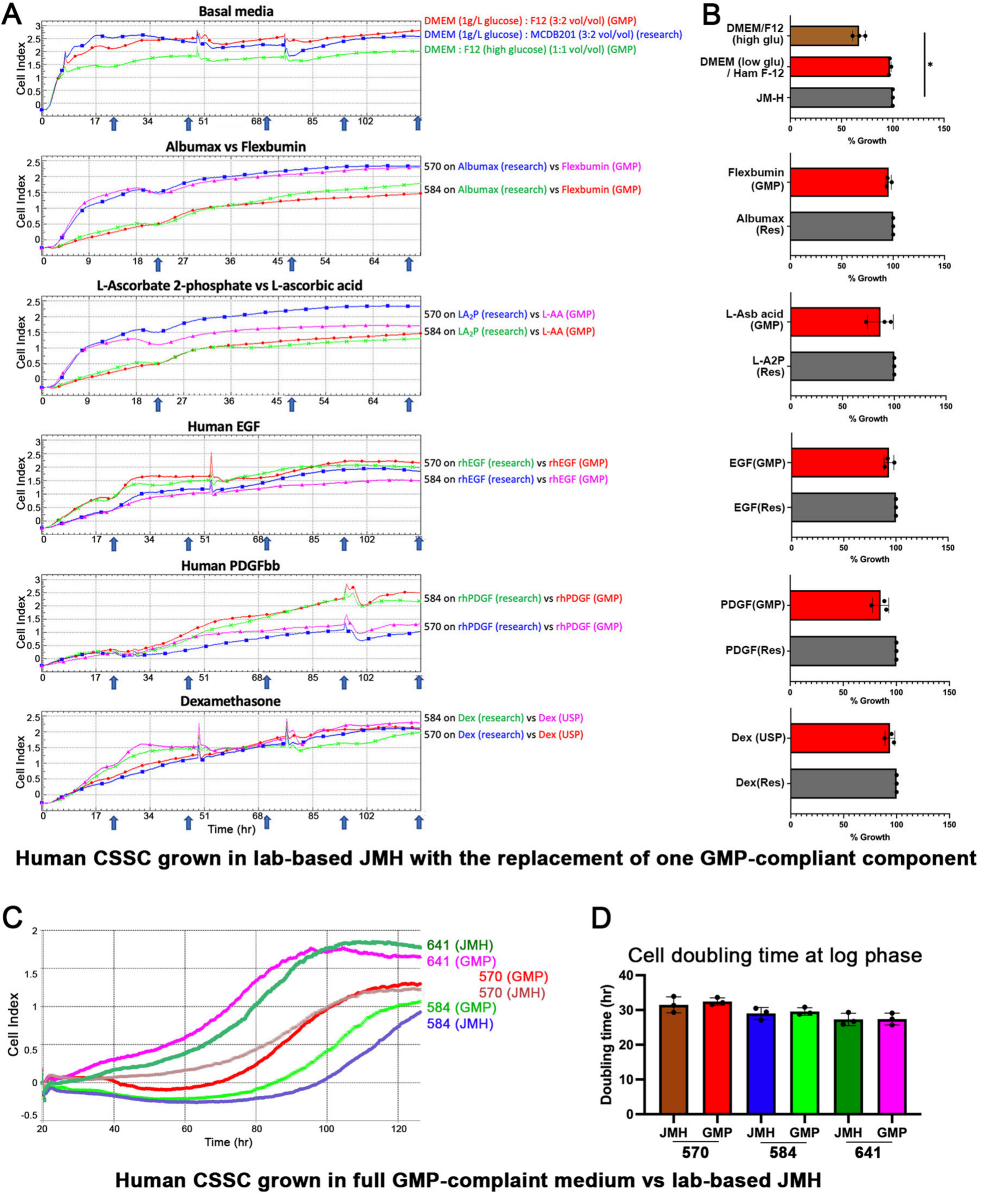
Optimizing GMP formulation of CSSC culture. **A** Growth kinetic profiles of donor CSSC batches in culture with JM-H medium with each research-grade component replaced by the corresponding GMP alternative. The numbers 570 and 584 represent donor corneal sources of CSSCs. Cell index profiles show the cell growth response up to 120 h. Blue arrows indicate 24-h intervals. The occasional interruptions are due to the pause for medium changes. The selected GMP substitutes showed similar culture outcomes as the research-grade reagents. **B** Percentages of cell growth recorded at 72 h show similar culture outcomes between each pair of GMP- and research-grade chemicals in CSSC propagation. *At the top profile indicates significant growth reduction of CSSCs cultured in DMEM/F12 (high L-glucose level) when compared to the mix of DMEM (low glucose) and MCDB201 in the laboratory-based JM-H medium (*P* < 0.05, one-way ANOVA). **C** Cell index profiles of CSSCs grown in the full GMP-compliant culture medium compared with the laboratory-based JM-H medium. **D** Cell doubling time at log phase illustrated similar CSSC growth in both media

Compared with JM-H which contains multiple components (Table 2), we used three donor CSSC batches at P1 and tested them with modified JM-H, where one component was replaced by the respective GMP partners. Their effects on cell stability and growth were monitored by xCELLigence which detects real-time electric impedance changes that represent cell viability, proliferation, and other cytopathic effects. Figure 2 shows the cell index profiles of donor CSSCs in diverse cultures with single GMP replacements. Comparable growth characteristics were confirmed when the cells showed no significant difference in cell indices at multiple time points (every 24 h up to 5 days) (arrows in Fig. 2A) and similar percentages of cell growth measured at 72 h after normalization with those from JM-H culture (Fig. 2B). The qualified GMP substitutes are listed in Table 2. A key finding was that the basal medium substituted by GMP-grade DMEM/F12 containing high glucose (4.5 g/L) was found to hinder CSSC growth with a drop of 32% at 72 h (P < 0.05; One-way ANOVA) (Fig. 2B). The mixture of DMEM (1 g/L D-glucose) with Ham-F12 (1.8 g/L D-glucose) in a 3:2 vol/vol ratio showed similar cell growth efficiency as the 3:2 volume mix of DMEM (1 g/L D-glucose) with MCDB201 in the original JM-H recipe. Hence, the basal media with higher glucose levels may not support the primary expansion of donor CSSCs. Figure 2C shows the cell index profiles of CSSCs grown in the full GMP-compliant culture medium compared with the laboratory-based JM-H medium. Three batches of primary CSSCs were run in triplicate and the growth profile did not exhibit any drastic difference. The cell doubling time at log phase further illustrated the similar growth of CSSCs in both media (Fig. 2D).

### Post-thaw recovery and characterization of cryopreserved CSSCs

**Fig. 3:**
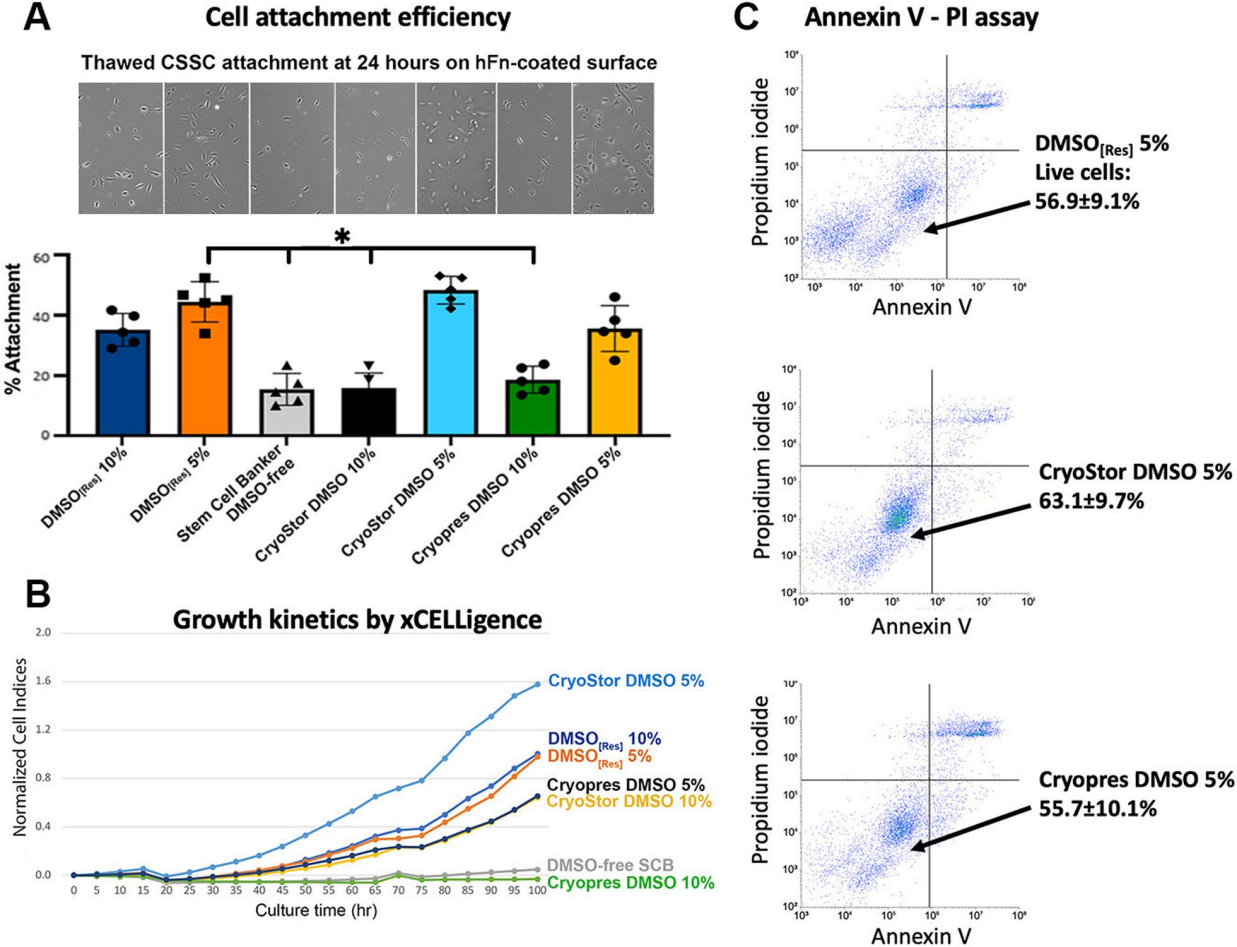
Comparative assessment of cryopreservation media for cultured CSSCs. **A** Cell attachment efficiency—thawed cells after one month of frozen storage in different cryopreservation media was seeded on hFn-coated culture surface. After 24 h, the attachment rates of cells stored in CryoStor DMSO 5% and Cryopres DMSO 5% were similar to the research-grade DMSO_[Res]_ (5%). Cell frozen in DMSO-free Stem-CellBanker, CryoStor DMSO 10%, and Cryopres DMSO 10% had poor cell attachment. **B** Cell index profiles of thawed CSSCs by xCELLigence. Cells in CryoStor DMSO 5% exhibited better growth kinetics. **C** Apoptosis assay by Annexin V-PI staining showed the percentages of live cells after frozen storage in 5% DMSO_[Res]_, CryoStor DMSO, 5% and Cryopres DMSO 5% were similar

Primary CSSCs cryopreserved in 5 to 10% DMSO (research-grade, Sigma; DMSO_[Res]_) were used as a reference for comparison with GMP-grade cryopreservation solutions—Stem-CellBanker (DMSO-free, SCB), CryoStor® CS10, and Cryopres™ DMSO (USP). After freezing for one and twelve months respectively, the post-thawed cells (n = 3 batches) were immediately examined for cell attachment on GMP-compliant hFn-coated surface for 24 h. hFn coating was selected due to its similar adhesion efficiency as the laboratory-based FNC coating described in previous section. Compared with cells cryostored with DMSO_[Res]_ at 10 or 5% in concentration (both showed 37.1 ± 5.4% and 43.8 ± 9.3% attachment rate), the use of CS5 by diluting CryoStor® CS10 with culture medium (1:1 vol/vol) and Cryopres™ DMSO at 5% yielded similar attachment efficiency (CS5 had 48.8 ± 3.5% and Cryopres™ DMSO at 5% had 32.7 ± 6.9%) (Fig. 3A). Cells cryopreserved with DMSO-free SCB, CS10, and Cryopres™ DMSO at 10% had significantly lower attachment rates (P < 0.05, compared to 5% DMSO_[Res]_; Mann–Whitney U test).

Cell apoptosis was examined using Annexin V and PI staining assay followed by flow cytometry. The post-thawed CSSCs from CS5 cryostorage exhibited better survival (63.1 ± 9.7% viable cells that were Annexin V and PI negative) (Fig. 3C), slightly surpassing the reference with 5% DMSO_[Res]_ (56.9 ± 9.1%) and Cryopres™ DMSO at 5% (55.7 ± 10.1%). Additional file 1: Fig. S3 displays the viable cell counts after storage with different cryopreservation media. Cell growth monitored by xCELLigence showed donor CSSCs cryopreserved with CS5 had a better revival and quicker exponential cell growth compared to other storage conditions (Fig. 3B).

### Comparison of expanded CSSCs derived from research versus GMP-compliant processing

Fresh donor corneal rims (*n* = 5) were divided into halves: one half was processed using the entire GMP-aligned procedures, and the other half using the laboratory-based protocol with JM-H culture. After anterior limbal stroma isolation and collagenase digestion, primary cells propagated at P1 were cryopreserved for one month, then thawed for P2 expansion and cell harvested for characterization.

#### Cell growth profile

**Fig. 4:**
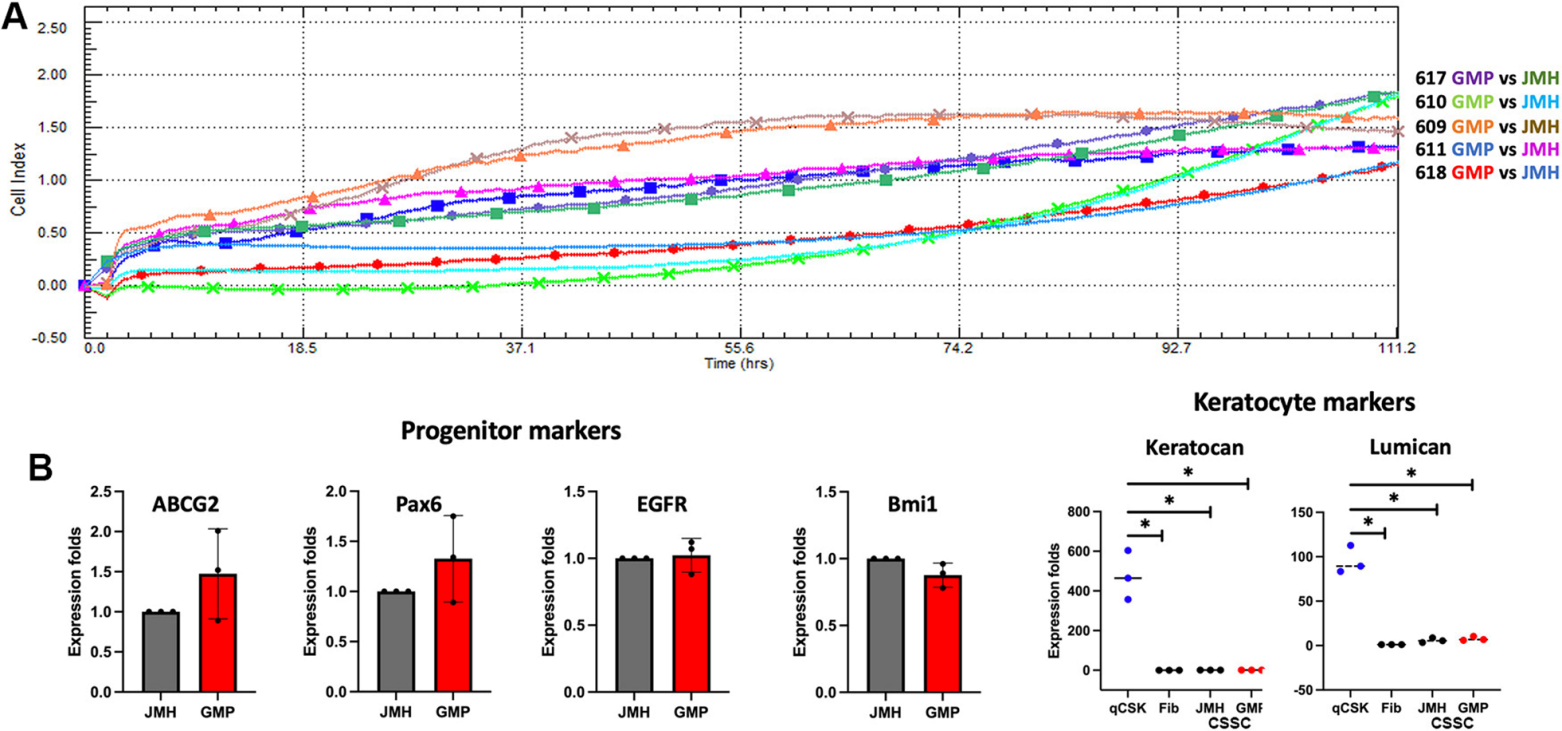
**A** Continuous growth kinetics of donor CSSC cultured in a pair-wise comparison of laboratory-based and GMP conditions. Both cell batches from donor corneas 610, 617 and 618 exhibited gradual propagation. **B** Similar expression levels of stem cell markers in GMP and JM-H raised CSSC. Both of them had significantly lower expression of keratocan and lumican, when compared to quiescent stromal keratocytes. SF—stromal fibroblasts were used as negative control for the reduced expression of keratocan and lumican. * P < 0.05, compared to quiescent keratocytes; one-way ANOVA, nonparametric

Continuous cell index collection up to 5 days revealed no difference between cultures under GMP versus JM-H conditions (Fig. 4A). Among these primary cultures, the 610, 617 and 618 cells grew continuously whereas two batches (609 and 611) underwent early senescence at P3 (Additional file 1: Fig. S4). The propagating 610 cell batch was subsequently named as GMP1.

#### Phenotypic evaluation

Both GMP and JM-H-raised donor CSSC expressed various progenitor markers (ABCG2, Pax6, Bmi1 and EGFR) at similar levels (Fig. 4B). When compared to quiescent stromal keratocytes, both GMP and JM-H-CSSCs showed attenuated expression of keratocan and lumican (*P* < 0.01, One-way ANOVA nonparametric).

#### Keratocyte differentiation in vitro

**Fig. 5:**
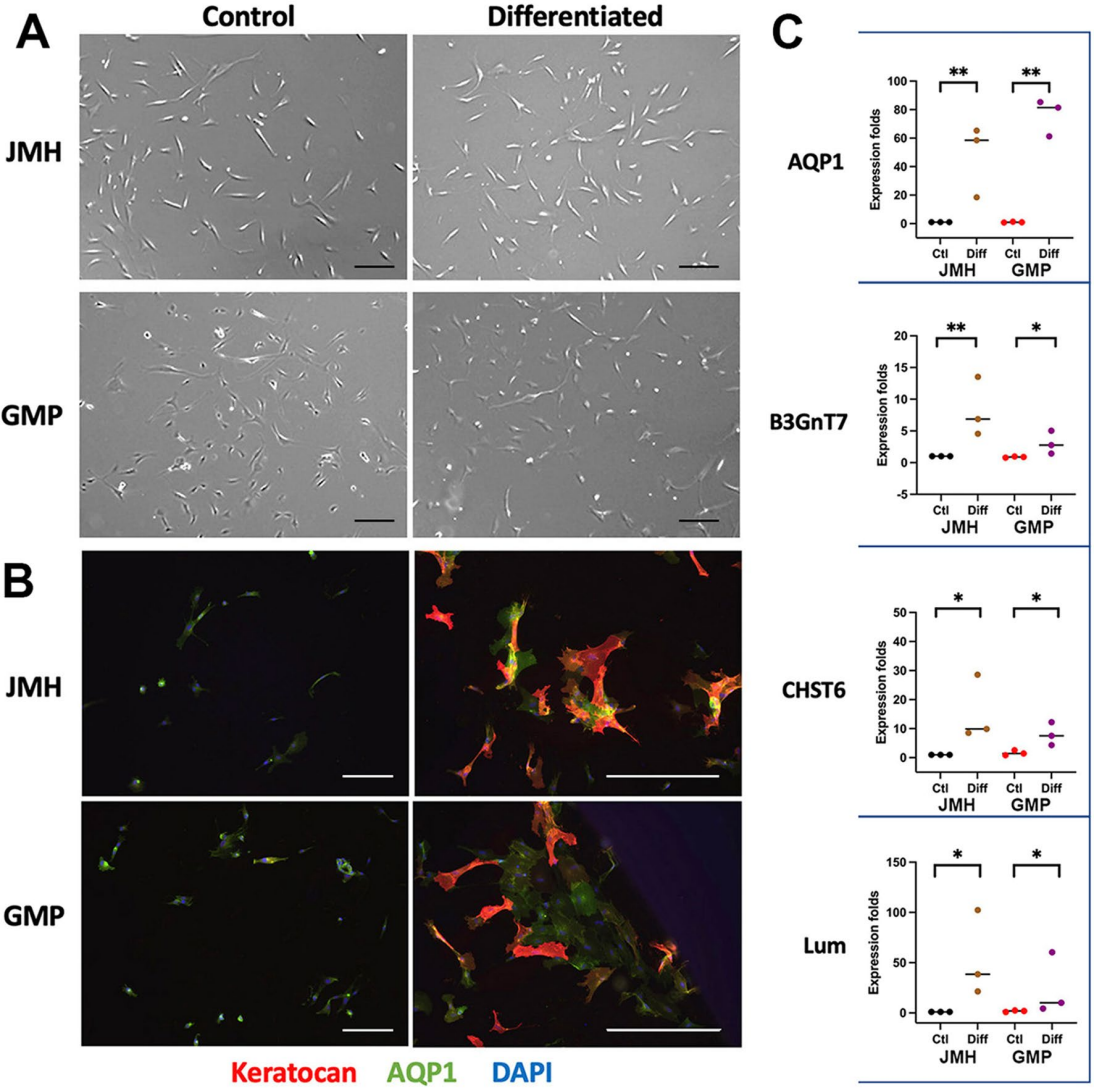
Keratocyte differentiation of CSSC under JM-H versus GMP cultures CSSC at P3 were cultured in a serum-free cytokine-supplemented condition (with bFGF and TGFβ3) for 7 days. **A** Typical dendritic morphology of keratocytes after induction. Scale bars: 50 µm. **B** Marked expression of keratocan (red) and aquaporin 1 (green) in the cytoplasm of differentiated cells. Scale bars: 50 µm **C** Similar response of laboratory-based and GMP-cultured CSSC in the expression of keratocyte markers, AQP1, B3GnT7, CHST6 and Lum. *P < 0.01; **P < 0.05, compared to undifferentiated CSSC; one-way ANOVA, nonparametric

Both laboratory-based and GMP-raised CSSCs (n = 3 batches), after a 7-day treatment with keratocyte differentiation protocol (refers to Method), showed typical keratocytes with dendritic morphology (Fig. 5A), marked upregulation of keratocyte-associated gene, keratocan, intracellularly by immunostaining (Fig. 5B). The differentiated cells also showed upregulated expression of aquaporin 1 (AQP1), lumican (Lum) and keratocan synthesizing enzymes B3GnT7 and CHST6 (Fig. 5C). All these changes were consistent between laboratory-based and GMP-cultured cells.

### Quality control (QC) assays

#### Stemness stability of CSSC—ABCG2 and nestin expression (ΔCT values)

**Fig. 6:**
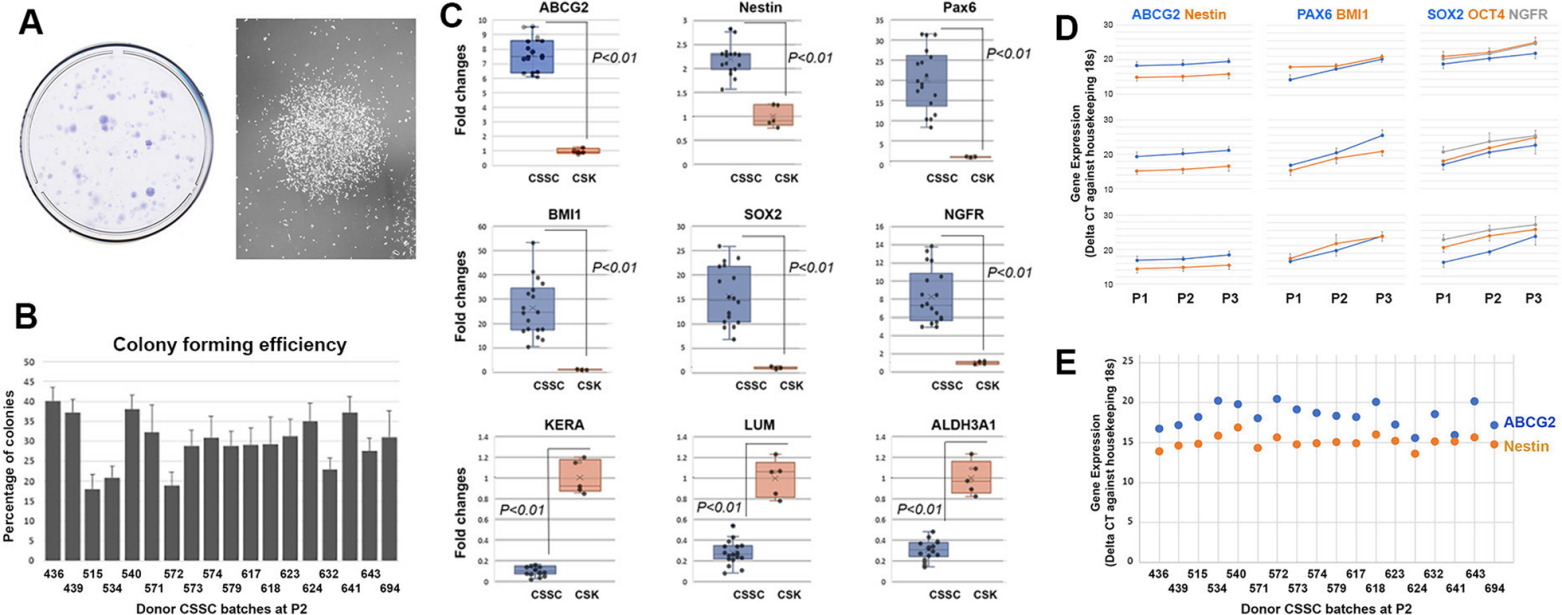
Donor CSSC culture and gene expression. **A** Clonal growth of P2 cells in JM-H medium. The crystal violet-stained colonies were imaged under brightfield microscopy. Phase-contrast micrograph showing a multicellular cluster (colony) of CSSC (HC641 at P2). **B** Colony-forming efficiency of 18 donor CSSC batches. The percentages of colonies (> 50 cells) were ranged from 18% to 41.1% and the mean CFU was 29.8 ± 6.5%. **C** The expression of stemness genes (ABCG2, nestin, Pax6, Bmi1, Sox2, NGFR) and keratocyte-specific genes (Kera, Lum and ALDH3A1) in cultured CSSCs (at P2) compared to quiescent keratocytes. **D** Passage-dependent changes of stemness genes in three representative CSSC batches derived from different donors. From P1 to P3, the cellular expression of ABCG2 and nestin remained consistent, whereas Pax6, Bmi1, Sox2, Oct4 and NGFR were gradually downregulated. **E** The expression patterns of ABCG2 and nestin were mostly consistent among 18 donor CSSC batches at P2

Donor CSSCs exhibited various progenitor features. Besides the formation of holoclones in culture (Fig. 6A), the primary CSSCs, in comparison with the quiescent keratocytes, showed elevated expression of progenitor genes (ABCG2, nestin, Pax6, Bmi1, Sox2 and NGFR), whereas the keratocyte-specific Kera, Lum, and ALDH3A1 were downregulated (Fig. 6B). In studying the stemness gene expression regarding cell passages, there were two expression patterns: ABCG2 and nestin were consistently expressed at P1, P2 and P3, but the expression of Pax6, Bmi1, Sox2, Oct4 and NGFR were gradually downregulated (Fig. 6D and Additional file 1: Fig. S5). At P2, ABCG2 and nestin showed a consistent trend of expression. The ΔCT of ABCG2 (normalized with housekeeping 18S) was about 16 to 20, whereas that of nestin was 12 to 16 (Fig. 6E, Additional file 1: Table S3). This indicated that primary CSSCs had a stronger expression of nestin than ABCG2, and the difference was maintained within 3 to 5 ΔCT values.

#### Anti-inflammatory potency—inhibition efficiency on a chronic pro-inflammatory macrophage-osteoclastogenesis

**Fig. 7:**
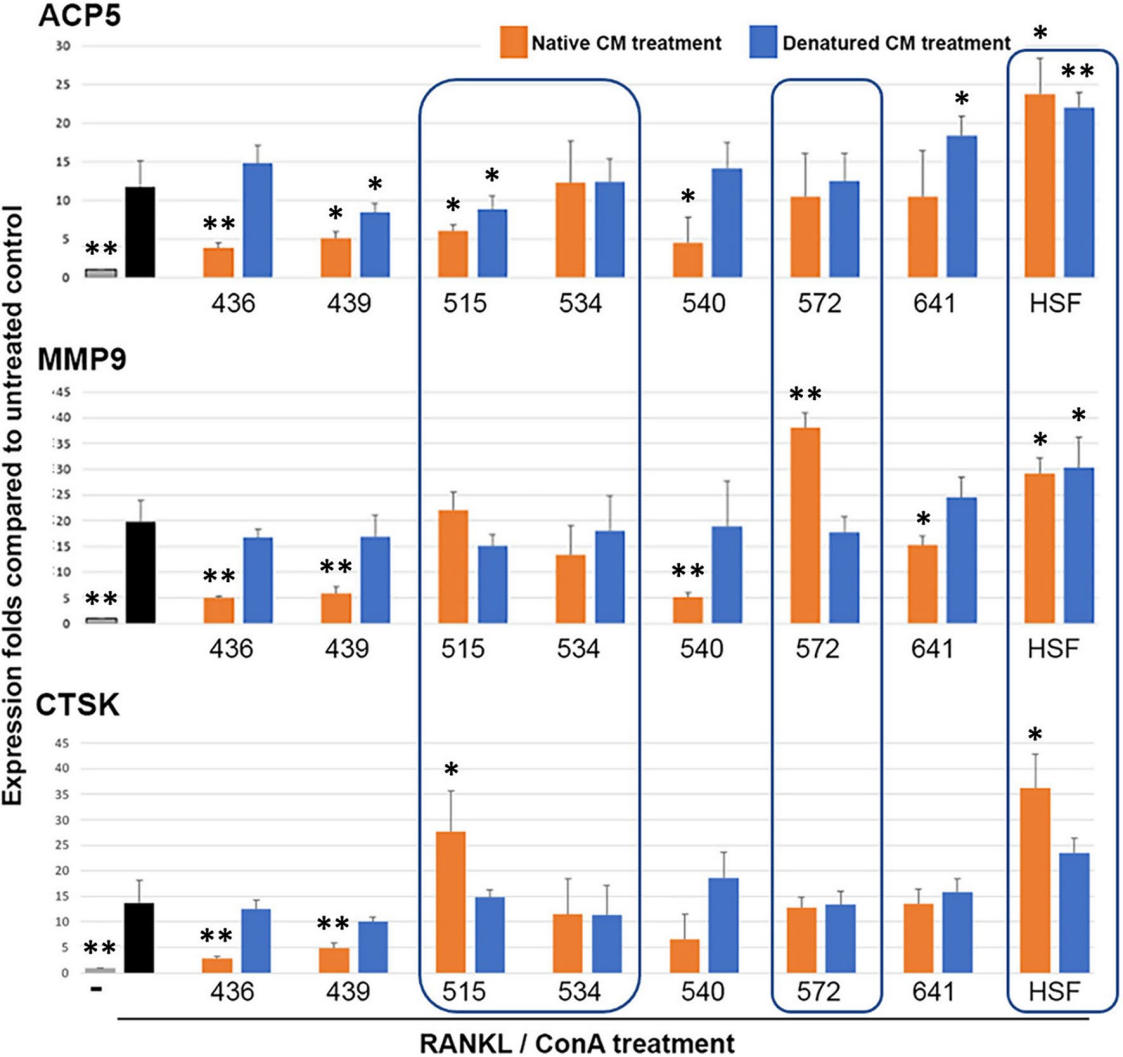
Anti-inflammatory property of primary CSSC at P2. Mouse RAW264.7 cells pre-incubated with heat-denatured CM concentrates showed upregulated expression of mouse ACP5, MMP9, and CTSK (blue colored) after RANKL/ConA stimulation for 5 days, similar to the controls (black colored). All three genes were downregulated after treatments using native CM (orange colored) from HC436, 439, 540 and 641, but not with CM from HC515, 534, and 572 (in bracket). The treatment with CM from human stromal fibroblasts (HSF) serves as a negative control without anti-inflammatory effect. Gray bars: naïve RAW cells; dark bars: RAW after RANKL/ConA treatment (without CM). *P < 0.05 and **P < 0.01 compared with RANKL/ConA-treated cells (Mann–Whitney U test)

The in vivo anti-inflammatory feature of CSSCs in mouse models of corneal wound correlates with the secretion of TSG-6 upon stimulation with TNFα in culture [12, 25]. Using a reported in vitro model of macrophage differentiation to osteoclasts under pro-inflammatory condition [26], we reported that the secretome or conditioned media from donor CSSCs with a better anti-scarring potential inhibited the cellular expression of ACP5, MMP9 and CTSK in mouse macrophage RAW264.7 under the induction to pro-inflammatory osteoclast lineage [16]. Here, CMconc (native versus heat-denatured) was applied at a constant 500 µg protein to RAW culture together with a 5-day treatment of RANKL peptide and ConA to induce osteoclast differentiation. Figure 7 shows the expression changes of mouse ACP5, MMP9 and CTSK, and the treatments with native CM from HC436, 439, 540 and 641 downregulated all three genes, when compared with the corresponding heat-denatured CMconc treatments. On the other hand, native CM from HC515, 534, and 572 did not suppress all three genes relative to the denatured CM treatments (brackets in Fig. 7). Data of all 18 donor CSSC batches are shown in Additional file 1: Fig. S6 and Table S3.

The rate of inflammation (RInflam) was calculated as the gene expression fold change ratio after the treatment of native versus denatured CMconc. The sum of RInflam is

ΣRInflam = ACP5(native/denatured) + MMP9(native/denatured) + CTSK(native/denatured).

Lower ΣRInflam values represent CSSC batches with higher anti-inflammatory potency.

#### A novel formula to calculate scarring index (SI) representing the anti-scarring potency of donor CSSC

We developed a formula integrating ΔCT(ABCG2), ΔCT(nestin) and ΣRInflam to calculate a numerical Scarring Index of CSSC batches to represent their anti-scarring potency.

$${\text{Scarring Index}}\left( {SI} \right) = \frac{{\left[ {2^{{DCT\left( {ABCG2} \right)}} + 2^{{DCT\left( {NES} \right)}} } \right]}}{m} + \frac{{2^{\Sigma RInflam} }}{n}\left( {\text{m and n are constants}} \right)$$

**Fig. 8:**
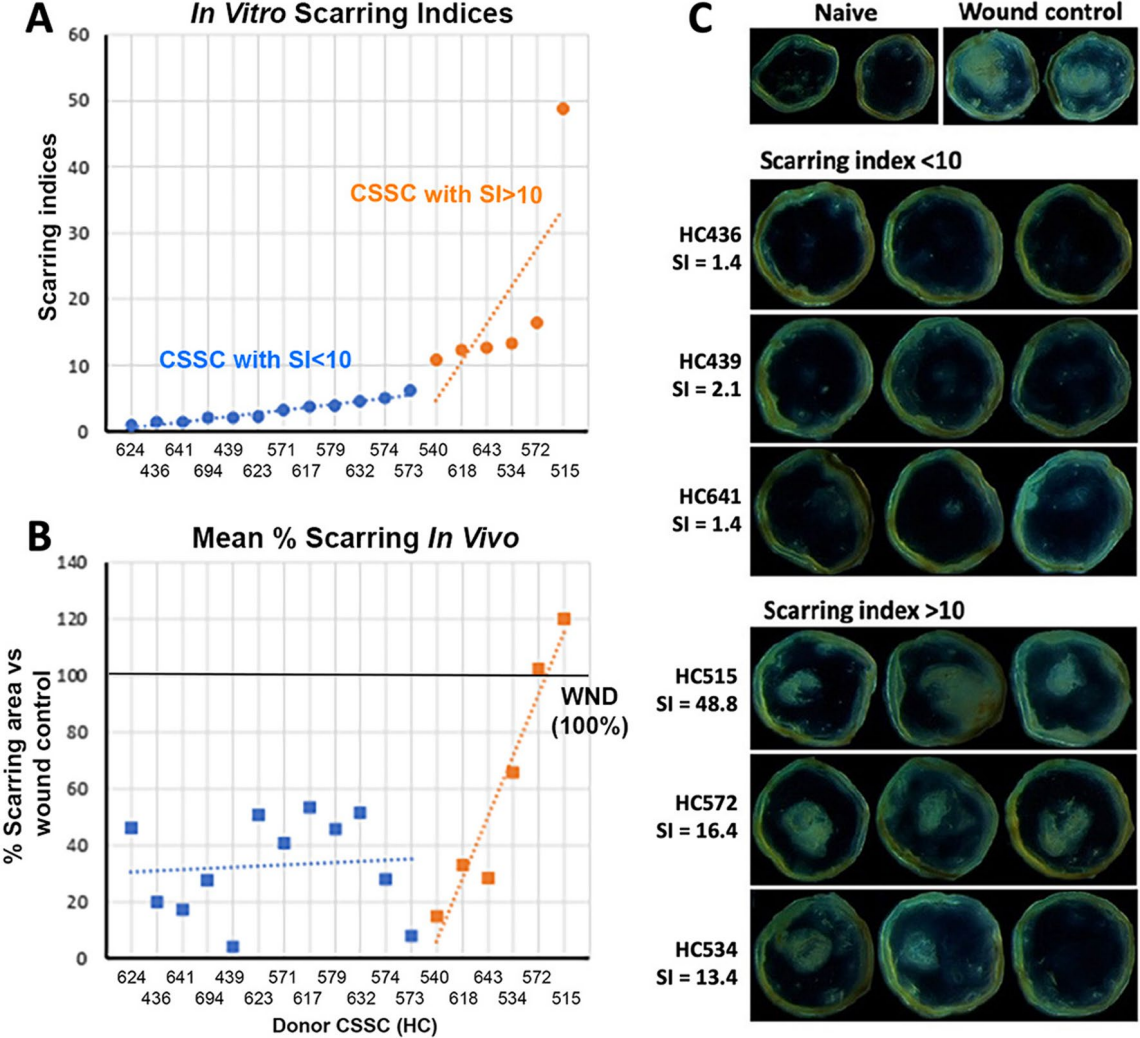
Scarring index (SI) represents the anti-scarring potential of donor CSSC. **A** Distribution of SI (round dots) calculated by our formula revealed donor CSSC (*n* = 18 cell batches). Labels on the x-axis indicate CSSC from different donors. CSSC with SI < 10 are colored in blue whereas cells with SI > 10 are orange-colored. **B** Mean percentages of scarring area (squares) inside mouse corneas after treatment with CSSC batches arranged with the same order as in **A** (*n* = 8 per group). Most cell treatments resulted in scar reduction, when compared with untreated wound control (100%, thick dark horizontal line), except HC572 and 515 with ineffective outcomes. Treatment with CSSC batches having in vitro SI < 10 (marked by the blue squares). The blue dotted regression line shows a stable regressive line at 32.7 ± 17.4% scar inhibition. For the treatment with CSSC having in vitro SI > 10, the regressive line showed an increasing trend of scar formation (orange dotted line). (**C**) Representative corneal images showing treatment outcomes with different CSSC having their respective SI values, compared with naïve and untreated wound controls

Figure 8A shows the SI calculated for 18 different donor CSSC batches (values in Additional file 1: Table S3). Twelve of them had SI values < 10 and the remaining six had higher SI. All these CSSC batches at P3 were tested in vivo for their anti-scarring effects using a mouse anterior corneal stromal injury model. Donor CSSCs (5 × 10^4^ cells) were applied to the acute wound created by Algerbrush ablation (n = 8 corneas per cell treatment). At day 14, the naïve corneas remained clear and untreated injured controls had intense scarring (Fig. 8C). CSSC-treated corneas showed different scar inhibitory outcomes. As an example, HC436, 439, and 641 treatments reduced scar formation, whereas HC515, 534, and 572 were moderate to ineffective in inhibiting corneal scarring. The overall results of corneal wound treatment with 18 different CSSC batches are displayed in Additional file 1: Fig. S7.

The percentages of scar area compared to wound control group were calculated. Figure 8B shows the mean percentages, which were substantially reduced after most CSSC treatments. The treatments with HC515 and 572 were incapable to control scarring. When we correlated this scarring outcome to the SI values, the cells with SI < 10 (blue round dots in Fig. 8A) had about 50% or less scar area (blue squares in Fig. 8B), compared to wound controls (dark horizontal line in Fig. 8B). The linear regression line (blue dotted line) showed a stable outcome of 32.7 ± 17.4% scar inhibition. On the other hand, treatment with cells having SI > 10 (orange round dots in Fig. 8A) showed moderate to ineffective (orange squares in Fig. 8B), though HC540, 618, and 643 that had SI marginally more than 10 show an opacity clearance. The orange regression line exhibited an increasing trend of scar formation. The treatment outcomes by these two groups of cells: SI < 10 versus SI > 10 showed statistically significant difference (*P* = 0.0312, Wilcoxon signed rank test).

#### Validation of SI indication using GMP-grade donor CSSCs

**Fig. 9:**
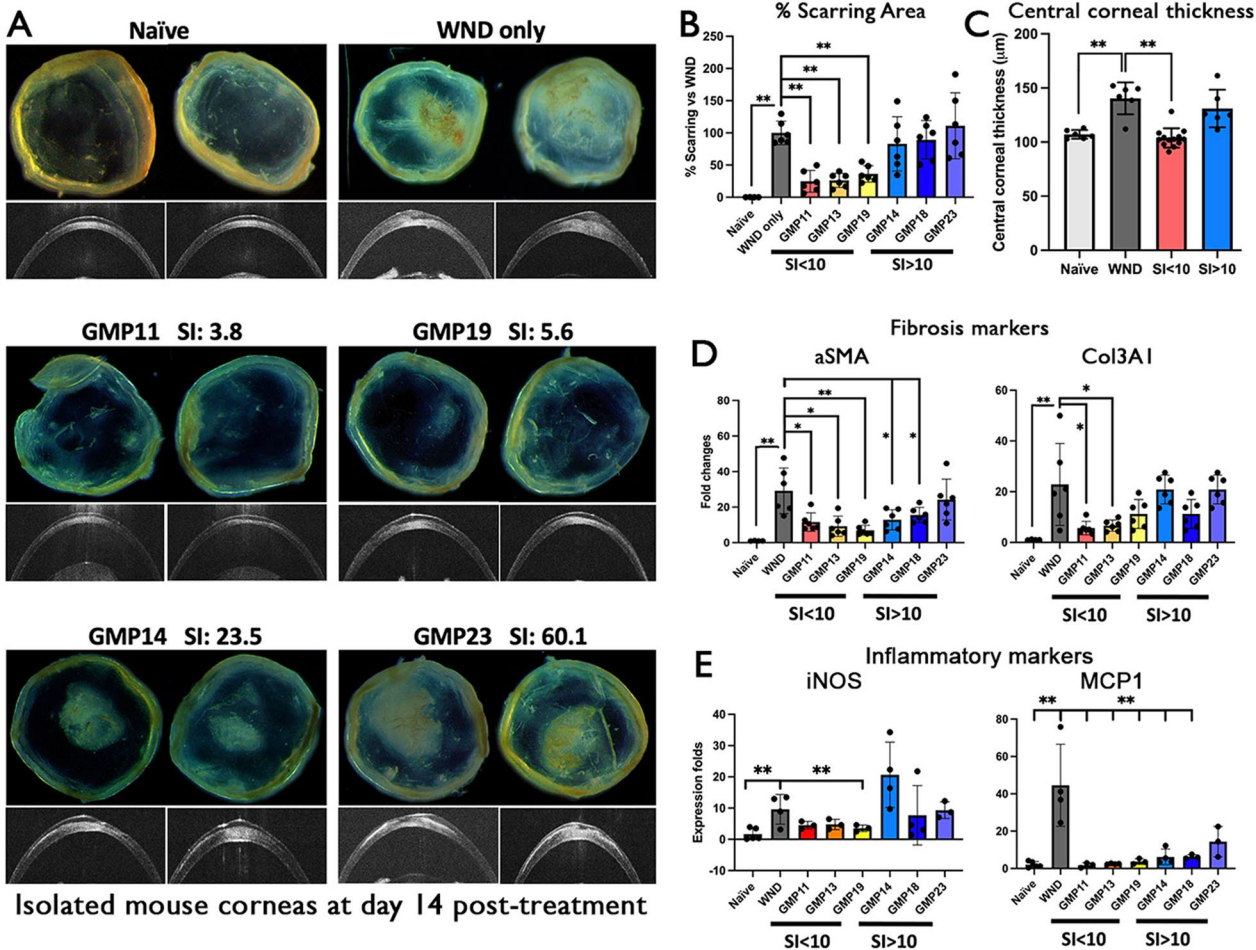
Treatment with CSSC_(GMP)_ on mouse corneas with anterior stromal injury to validate the SI prediction of anti-scarring potency. **A** Mouse corneal images showing the scarring outcomes at day 14 post-treatment with CSSC_(GMP)_ having different SI values. Cells with SI < 10 (middle row) showed strong scar inhibitory effect while cells with SI > 10 (bottom row) were ineffective to control scar formation. Anterior segment OCT images show the cross-sectional corneal changes. **B** Percentages of scarring area—treatment of CSSC_(GMP)_ with SI < 10 had significant scar inhibition, whereas injured corneas treated by CSSC_(GMP)_ with SI > 10 showed similar scarring as the untreated wound controls (WND). (**C**) Central corneal thickness (CCT) measured using ASOCT images (in panel A)—the scar-reducing outcome by CSSC_(GMP)_ with SI < 10 significantly reduced corneal thickening due to stromal injury (WND). **D** and **E** Significant downregulated expression of fibrosis (αSMA, Col3a1) and inflammatory genes (iNOS and MCP1) after treatment with CSSC_(GMP)_ having SI < 10 when compared to WND. *P < 0.05, **P < 0.01 (one-way ANOVA, nonparametric)

To validate the in vivo anti-scarring prediction using the novel SI calculation and to provide insight into the clinically compliant cell products, we tested six different GMP-raised donor CSSC batches at P2 with the in vitro stemness stability and osteoclastogenesis assays, and cells at P3 for corneal wound treatment in mice. Topical application of prototype CSSC_(GMP)_ with SI < 10 in a drop of fibrin gel to the anterior stromal wound successfully inhibited corneal scarring down to less than 50%, when compared to the wound control without cell treatment (set as 100% scarring). The treatment with GMP11 (SI = 3.75) effected with 12.2 ± 15.8% scarring, GMP13 (SI = 6.84) with 28.1 ± 14.1% scarring, and GMP19 (SI = 5.61) with 31.4 ± 14.5% scarring (Fig. 9A and B). All these treated corneas were significantly less opaque and had reduced percentages of scar area than the untreated wound control (**P < 0.01, Mann–Whitney U test; n = 6 per treatment). The other three CSSC_(GMP)_ batches with SI > 10, however, did not inhibit scar formation: GMP14 (SI = 23.5) showed 79.4 ± 45.1%, GMP18 (SI = 45.9) had 76.4 ± 28.4%, and GMP23 (SI = 60.1) resulted in 111.1 ± 51.1% scarring, which were insignificant different with wound controls. The anti-scarring outcome by CSSC_(GMP)_ with SI < 10 was accompanied with a recovery of central corneal thickness resembling the naïve corneas (Fig. 9C).

Mouse corneas inflicted by stromal injury had significant upregulation of fibrosis markers (αSMA and Col3A1) (Fig. 9D) and inflammatory genes (iNOS and MCP1) (Fig. 9E). After treatment with CSSC_(GMP)_ having SI < 10, the expression of these markers was significantly reduced, whereas those treated with CSSC_(GMP)_ having SI > 10 had inconsistent and insignificant changes.

## Discussion

Our work firstly established a GMP-compliant protocol covering donor limbal stromal tissue processing, enzymatic digestion, primary CSSC culture, and cryopreservation, to advance the CSSC therapy for clinical trials in patients with corneal scarring. By comparatively assessing the GMP-compliant reagents with the corresponding research-grade chemicals used in our laboratory-optimized protocol (non-GMP-compliant) [6, 8–13, 15, 16, 22, 24], we finalized a GMP formulation of culture medium and presented a comprehensive evaluation of GMP-compatible CSSC [CSSC_(GMP)_] propagation, as a first optimization step toward clinical-grade cell manufacturing. The stem cell phenotypes and the characteristic capability to differentiate into stromal keratocytes were confirmed for CSSC_(GMP)_, which was similar to the research-grade cells [CSSC_(Res)_]. More importantly, we developed a Quality Management System using in vitro QC assays by examining the stemness stability (ABCG2 and nestin) and the anti-inflammatory ability of CSSCs. Both parameters were incorporated using a novel formula to calculate a numerical Scarring Index (SI) for each CSSC batch. Correlating with the in vivo scar inhibitory outcomes utilizing a mouse model of anterior stromal injury, we demonstrated that donor CSSCs with SI < 10 had a predicted 50% scar reduction potency while cells with SI > 10 were ineffective to control scarring and they should be excluded for patient use. After the validation using CSSC_(GMP)_, our QC testing and SI calculation proved to be suitable for evaluating the potency of cell products in a scar-reducing corneal wound healing.

Cell therapies are actively being pursued in the hope of treating a broad range of conditions and intractable diseases. One significant component of FDA cGMP is to develop QC to ensure the consistency and quality of these therapies. This includes in-process testing, release testing, and stability testing of IND products. Also the analytical methods used for testing must be validated. Given that each IND production process is unique, an internally developed and validated QC metric is critical to generate Quality Assurance for the IND production. Conversely, therapeutic cell products not manufactured under a well-validated and characterized process may inflict serious adverse effects on patients. If not controlled, this can also damage the reputation of the legitimate stem cell research and therapy. It is unlikely to have “one-size-fits-all” set of procedures for stem cell production, like the use of a proprietary culture medium for distinct types of stem cells and generic QC assays to assess the product quality. The current study provides the first unique set of QC metrics that correlate with in vitro characteristics of CSSCs that would generate the desired therapeutic effect of corneal healing. These cellular attributes will still require a longitudinal monitoring and evaluation under a thoroughly designed stability program as CSSC_(GMP)_ products are to be cryopreserved as master cell banks as an allogeneic, off-the-shelf product in the future.

Building upon our reports of propagating primary donor CSSCs with JM-H medium protocol, we identified the research-grade components in the medium formulation and replaced with the equivalent clinical-grade GMP-compliant reagents. This refinement reduces the potential threat of pathological effects and immunological reactions following cell transplantation. The development of a GMP-compliant culture system is necessary in moving forward to the next stage of clinical trials, of which the national and local regulatory bodies emphasize the treatment safety and efficacy. Initial studies of research-grade reagents as compared to their respective GMP alternatives were performed for each key process. Those reagents with outcomes that were as good, if not better, were to be included in the final GMP-aligned formulation for human CSSC culture. Our work was started with the limbal stromal tissue digestion to release CSSCs to establish primary culture. Tissue dissociation using GMP NB6 collagenase (Nordmark) was comparable to the research-grade collagenase A (Roche) and viable cell yields revealed by trypan blue dye exclusion assay were similar (Fig. 1). In contrast, the digestion with Celase (Worthington) gave significantly reduced cell viability. The use of ECM for primary cell attachment to initiate a two-dimensional CSSC culture demonstrated that the cell attachment efficiency on GMP-grade hFn (R&D) was comparable to FNC-coated culture surface routinely used in our laboratory-based cultures. The coating with hLn-521, however, did not enhance cell adhesion.

A critical observation was made for the basal culture medium. We found that the commonly used GMP-compliant DMEM/F12 did not fully support donor CSSC growth due to its high content of D-glucose (4.5 g/L in concentration). In contrast, a mix of GMP-made DMEM (1 g/L glucose) with Ham-F12 (1.8 g/L glucose) in a 3:2 vol/vol (giving a final 1.12 g/L glucose) showed similar cell growth efficiency as the 3:2 volume mix of DMEM (1 g/L glucose) with MCDB201 (1.44 g/L glucose), resulting in a final glucose level of 1.18 g/L, as we used in the original JM-H recipe. Hence, a culture medium with correct glucose level could favor CSSC growth in vitro. Supplementation with a low level of serum (2%) stimulates CSSC proliferation [27]. However, the poorly defined composition and batch-to-batch variability of sera have complicated the optimization step. In our laboratory-based protocol, the use of pooled human serum has been optimized, and we continue to employ this condition for CSSC_(GMP)_ cultures. Autologous serum from patients harvested to yield CSSCs for transplantation could avoid the possible influence of inter-individual’s variability. The detailed effects on cell responsiveness and product consistency need to be investigated in future studies. Our preliminary work tested commercial GMP-grade AB human sera from Akron Biotech; however, it failed to yield successful CSSC cultures (data not shown). Variable effects of AB sera have been reported in its support of growth of some MSC types and dental pulp-derived stem cells [28, 29] as well as inhibition of growth of primary cardiac progenitors [30].

High cell viability after cryopreservation allows the distribution and transportation of the propagated CSSC products to secondary sites for clinical use and mitigates the current aspect of donor material shortage. The laboratory-based protocol uses a freezing solution containing 5 to 10% DMSO_[Res]_ in JM-H medium. In an early attempt to freeze down CSSCs with a DMSO-free, xeno-free Stem-CellBanker (Amsbio) with direct freezing at -80 °C per manufacturer’s instruction, the cell viability, attachment, and growth kinetics dropped significantly after frozen for either 1 or 12 months, compared to the conventional DMSO_[Res]_ freezing. This indicates that DMSO-based cryopreservation may provide a mechanistic advantage to encourage post-thaw viability and functional recovery for the cryopreserved human CSSCs. Subsequently, the screening of cell survival and growth after cryopreservation with different GMP-made DMSO-containing solutions have identified that GMP-grade CryoStor® CS10 and USP-grade Cryopres™ DMSO at 10% were also unable to maintain a high yield of viable CSSCs. In contrast, by diluting CS10 or Cryopres® DMSO to a concentration of 5% with CSSC culture medium, the post-thawed cell viability, attachment to the culture surface and cell growth kinetics were improved and comparable to 5 or 10% DMSO_[Res]_. At low density seeding, the CSSC revived and exhibited clonal growth with a homogenous population. Such preparation of diluted GMP-grade DMSO suggests that certain ingredients inside CSSC culture medium could be critical for cell viability and growth, and they are essential during cryopreservation. This also reflects that stem cell media (other than the optimized CSSC growth medium) may not be favorable to sustain CSSC survival and expansion. The success of optimizing cryopreservation makes possible the distribution of cell products to worldwide locations for the purpose of CSSC therapy. The extent of CSSC survival after freezing differs among donors, and the clinical application of cryopreserved CSSCs for transplantation in terms of cell functionality will have to be evaluated and characterized in future studies.

Combining the qualified GMP reagents to a final GMP-compliant formulation, we propagated an independent series of CSSCs. From the same donor cornea, half rim of the limbal stroma was GMP processed, and the other half followed the laboratory-based protocol. The CSSC_(GMP)_ at P2 expressed stem cell marker genes (ABCG2, Pax6, EGFR, and Bmi1), and similar low levels of keratocyte-specific markers (Kera and Lum) as the CSSC_(RES)_ from the same donor. In our study, out of five sets of CSSC_(GMP)_, three batches continued to propagate at least up to P3, while two cultures underwent early senescence, which is commonly observed for primary cell cultures. Such growth reduction was seen for both CSSC_(GMP)_ and CSSC_(Res)_ from the same donor, indicative of donor specificity. Further analyses of cellular and genomic alterations, such as chromosomal aberrations by karyotyping, or their structural variants (e.g., copy number variation and chromosomal rearrangement by chromosomal-based microarray analysis), as well as global gene expression profiling by next-generation RNA sequencing will be performed in a separate study.

Next, we established QC testing which is a regulatory requirement when CSSC products are launched for clinical use. The cell characteristics, functionality, and potency, though being processed with the GMP-aligned protocols, can potentially vary from donor to donor. Hence, there is a critical need to control the final product quality and set up specifications for product release. The limited amount of in-process materials during clinical-grade cell production presents a logistical challenge in performing a full analytical assessment of CSSC products. Hence, understanding and pinpointing at the cells’ most notable effect in correcting tissue defects will help us identify appropriate in vitro assays for quality evaluation. This also refers to the product potency, which represents a mean to examine the product quality and may relate to but not exactly define the product’s clinical efficacy. Besides the batch-to-batch variability that occurs from different donor corneas, the variability can also occur in the scale-up manufacturing process due to differences in equipment, reagents, and supplies.

It is imperative that the propagated CSSCs should retain stem cell features. Cell products that have undergone spontaneous differentiation or fibroblastic changes should be disqualified for clinical use. Hence, we opted to assess the cellular expression of stemness genes and identified stem cell markers that are invariably expressed during the early CSSC culture from P1 to P3. This narrow time window is due to the conventional protocol that primary CSSCs be expanded from P1 to P3, and the cells at P3 (about 70–80 population doublings from initial cells) are used for wound treatment [6, 8, 9, 24]. Cells expanded beyond P3 could reach replicative senescence or exhibit unstable stem cell properties [23]. Among the reported stemness genes, our work showed that mRNA for *PAX6*, *BMI1*, *SOX2*, and *OCT4* genes were markedly reduced when CSSCs were expanded from P1 to P3. These downregulated genes are known to play roles in early ocular development and embryonic neural crest maintenance [31]. On the contrary, the more consistent expression of *ABCG2* and *NESTIN* across P1 to P3 suggests that the expanded CSSCs attain certain levels of general stem cell characteristics. ABCG2 protein is an ABC (ATP-binding cassette) transporter, also known as breast cancer resistance protein (BCRP), is often expressed in stem cells [32]. In our early report, human CSSCs with *ABCG2* expression exhibited a specific “side population” of cells able to efflux toxin or dye (e.g., Hoechst 33,342) when analyzed by flow cytometry, and this population was lost after treatment with verapamil [10]. Continual expression of this property confers CSSC the ability in xenobiotic protection, multidrug resistance, and detoxification [32]. By immunohistochemistry, elevated levels of ABCG2 expression are reported in syncytiotrophoblasts of the placenta, liver, adrenal gland, sebaceous glands, testes, and uterus [33, 34]. Many of these ABCG2-positive cells were found to have a secretory role, suggesting ABCG2 to have a role beyond stem cell protection. Whether ABCG2-expressing CSSCs actively produce and release factors attributing to potential autocrine or paracrine activity remains to be investigated.

In addition, CSSCs contain immunomodulatory activity consistent with MSC from other sources [23]. As demonstrated from pre-clinical studies, CSSC treatments suppressed early neutrophil infiltration in response to corneal trauma [12] and attenuated tissue inflammation, which is essential to the development of opaque scar tissue in the injured corneas. The CSSC’s immunomodulatory effect was found to be associated with microRNA expression, specifically hsa-miR-29a and 381 [15, 16]. These miRNAs were identified when investigating CSSC-secreted soluble factors that modulated the pro-inflammatory osteoclast-like differentiation of RAW macrophages after RANKL and ConA treatment. This assay has thus illustrated that good quality CSSC should possess anti-inflammatory potency. We employed this potency feature as a quantitative measure to monitor CSSC’s biological effect. Using secretome or conditioned media from CSSC cultures, we identified a differential effect of native versus heat-denatured CM samples on the induced osteoclast gene expression of RAW macrophages. By downregulating all three osteoclast genes (ACP5, MMP9, and CTSK) and calculating their expression fold change ratio between native versus heat-denatured sample treatment, which is referred as the rate of inflammation (RInflam), we identified the anti-inflammatory potency of different CSSC batches. Such screening of anti-inflammatory activity is highly feasible as this avoids using the limited cell product, and the medium sampling can be obtained during the scale-up cell manufacturing as in-process testing or at the end of production prior to the release of cell product for clinical use.

With the novel formula incorporating both the expression of stemness genes ABCG2 and nestin (ΔCT values after normalization with the housekeeping 18S) and the RInflam of three osteoclast genes, to calculate the Scarring Index (SI), we found that the healing potency of CSSCs can be predictable. The mouse model of mechanical anterior stromal injury created by Algerbrush burring has been deemed as the most effective way to mimic the traumatic injury in humans [35]. Inflicted by the rotating head of Algerbrush, the localized corneal wound with damaged Bowman’s membrane exhibits pronounced keratocyte apoptosis and activation of repair-type fibroblasts [36, 37]. Effected by the pro-fibrotic cytokines (e.g., TGFbß and platelet-derived growth factor), the generation of myofibroblasts contributes to the overproduction of abnormal ECM, which deposits in a disorganized manner and with excessive tissue contraction, resulting in opacities and scar formation. We demonstrated using 18 different donor CSSC batches that cells with SI < 10 were effective to reduce scar formation by around 50%, when compared with untreated wound controls. In contrast, cells with SI > 10 had moderate to ineffective control of scar formation. This potency prediction was validated with CSSC_(GMP)_ (*n* = 6 batches). Mouse corneas treated with CSSC_(GMP)_ (SI < 10) showed reduced opacities and tissue expression of scar-related fibrosis genes (Col3a1 and αSMA), supporting our notion that CSSC therapy rescued corneal scarring. Further work will include the use of additional CSSC_(GMP)_ batches with SI calculation to test their effectiveness on scar-reducing healing using other reported corneal injury models (e.g., alkali burns and laser keratectomy).

One of the limitations of our work is that some CSSC batches with SI greater than 10 could deliver a proper anti-scarring effect in vivo, e.g., the treatment with HC540, 618, and 643 resulted in ~ 50% scar reduction. Though these cells showed scar inhibition, we set the threshold for cells with SI < 10 to ensure the best quality of CSSC product for clinical applications. In addition, both QC assays (stemness stability and anti-inflammatory potency) involve lengthy laboratory work and utilization of other cell type (RAW264.7 cells), which could introduce variations in the assay results. Simple assays with readily detectable outcome could be favorable for FDA IND applications.

## Conclusions

Our work has translated the CSSC therapy and research outcomes obtained from a laboratory-based setting to clinically relevant and GMP-compliant protocols in order to ensure the cell product safety and quality. We showed the robustness of human CSSC propagation using a GMP-aligned medium formulation and protocol, and the characterization studies of CSSC_(GMP)_ showed the cell features that are as good, if not better than CSSC generated using the research formulation. More importantly, the QC assays and calculation of SI values have provided an important indication to predict the healing and anti-scarring potency of the cells. This novel method of in vitro–in vivo correlation to obtain a predictive factor from in vitro assays to indicate the in vivo potency and effectiveness could apply to other cell-based therapies or pharmacological treatments.

### Supplementary Information

**Additional file 1.** Supplementary figures and tables.

## Data Availability

All data are included in the text and supplementary materials. Data details are available from the corresponding author on request.

## Abbreviations

ABCG2: ATP-binding cassette subfamily G member transporter 2
ACP5: Alkaline phosphatase
bFGF: Basic fibroblast growth factor
CI: Cell Index
CM: Conditioned media
CMconc: Conditioned medium concentrate
Col3: Collagen III
CP: Cryopres™
CS: CryoStor®
CSSC: Corneal stromal stem cell
CTSK: Cathepsin K
DMSO: Dimethyl sulfoxide
EV: Extracellular vesicles
FDA: Food and drug administration
FITC: Fluorescein isothiocyanate
FN: Fibronectin
GMP: Good manufacturing practice
IND: Investigational new drug
Kera: Keratocan
Lum: Lumican
MMP: Matrix metalloproteinase
MSC: Mesenchymal stem cell
PBS: Phosphate buffered saline
PCR: Polymerase chain reaction
PI: Propidium iodide
QC: Quality control
rhEGF: Recombinant human epidermal growth factor
rhPDGF: Recombinant human platelet-derived growth factor
RInflam: Rate of inflammation
SCB: Stem-CellBanker
SD-OCT: Spectral domain optical coherence tomography
SI: Scarring index
αSMA: α-Smooth muscle actin
TGF: Transforming growth factor
TNC: Tenascin C
TSG-6: Tumor necrosis factor α-stimulated gene 6
