# Supplementary material for: Good manufacturing practice production of human corneal limbus-derived stromal stem cells and in vitro quality screening for therapeutic inhibition of corneal scarring
Source: Stem Cell Res Ther. 2024 Jan 8;15:11. doi: 10.1186/s13287-023-03626-8 (PMC10773078; doi:10.1186/s13287-023-03626-8)
Supplement: Supplementary file 1 — Additional file 1. Supplementary figures and tables. [file 13287_2023_3626_MOESM1_ESM.pdf]

Good manufacturing practice production of human corneal limbus-derived stromal stem cells and in vitro quality screening for therapeutic applications

Mithun Santra, Michael Y.S. Hsu, Moira L Geary, Elizabeth Rubin, Martha L Funderburgh, Christine Chandran, Yiqin Du, Deepinder K Dhaliwal, Vishal Jhanji, and Gary Yam

Supplemental Materials

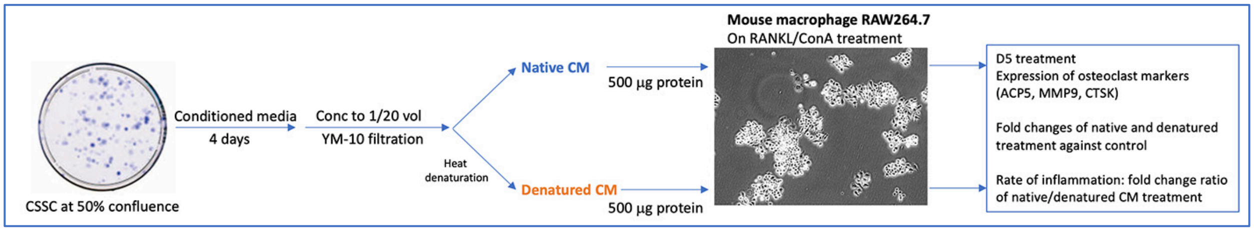

Supplementary Figure S1. Chronic osteoclastogenesis assay. CSSC-derived conditioned media (CM) were concentrated and quantified for total protein content. Both native and heat-denatured CM samples (500 □g protein) were added to RAW264.7 cultures followed by RANKL/ConA treatment for 5 days to induce osteoclastogenesis. The cellular expression changes of ACP5, MMP9 and CTSK caused by CM treatment (native versus denatured) were assayed, and the ratio of expression fold changes were used in Scarring Index calculation.

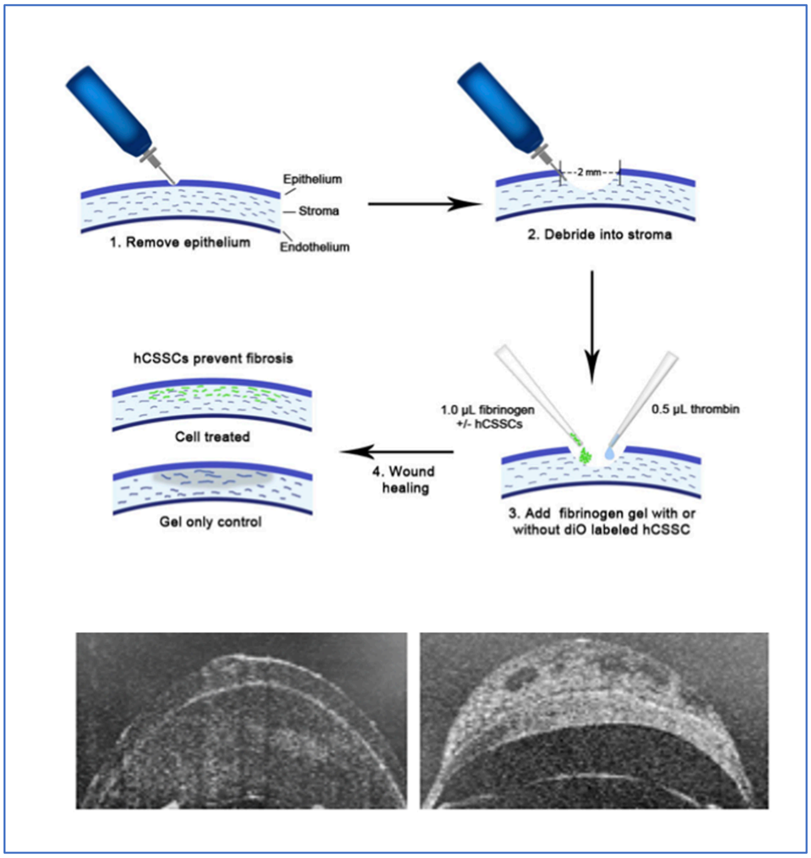

Supplemental Figure S2. A mouse model of corneal anterior stromal injury. Using an Algerbrush mechanical burring to create an epithelial wound of 2 mm diameter, the injury disrupted the Bowman's membrane and produced a superficial stromal wound of 10-20 □m depth. Immediately after injury and washes, cells were applied in a fibrin gel (fibrinogen and thrombin). OCT pictures at the bottom panel show the cross-sectional cornea after injury and post-treatment with the cell drop located on the corneal surface.

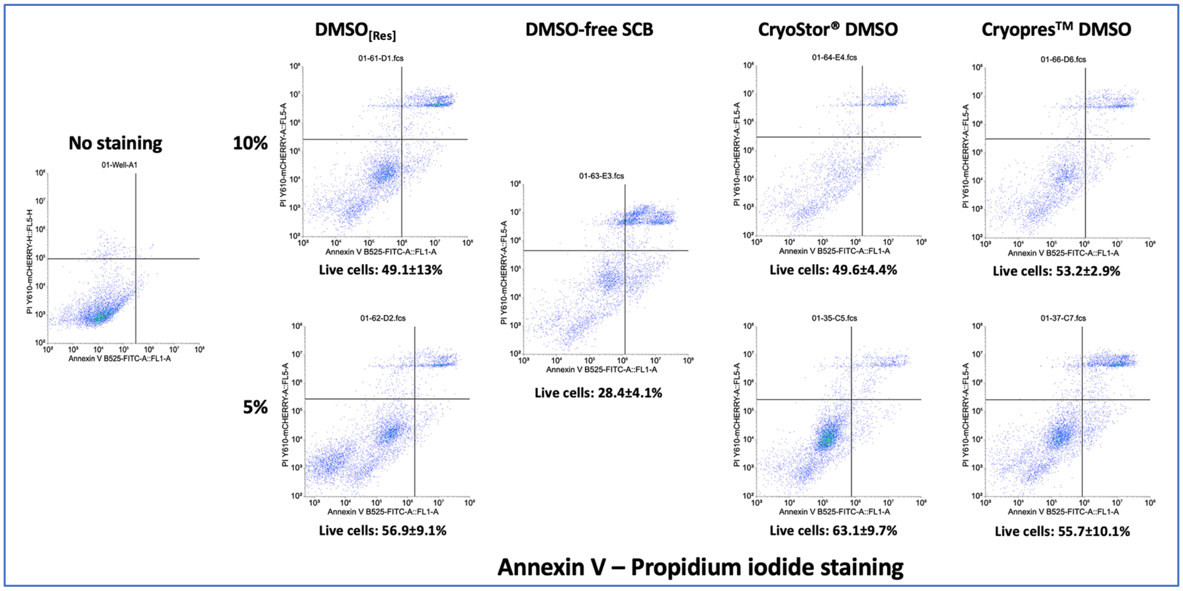

Supplemental Figure S3 Apoptosis assay by annexin V-PI staining followed by examination with flow cytometry showed the percentages of live cells frozen in different cryo-preservation media. The conditions of 5% $DMSO_{[Res]}$, CryoStor DMSO 5%, and Cryopres DMSO 5% gave the similarly high cell viability.

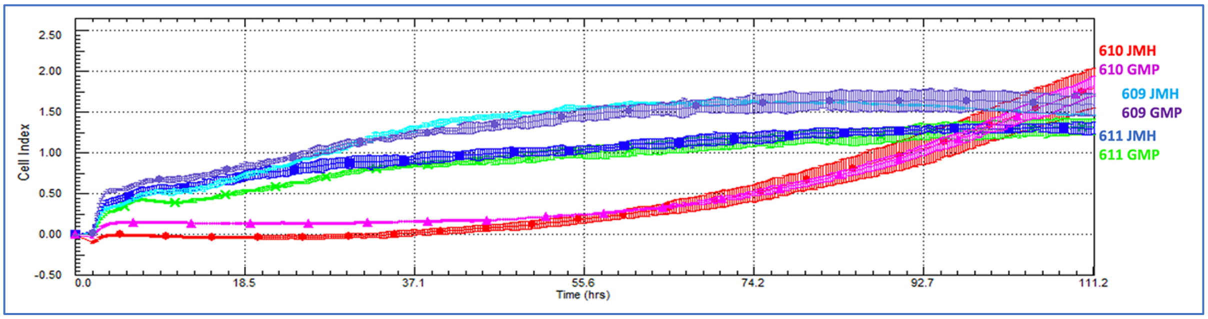

Supplemental Figure S4. Continuous growth kinetics of donor CSSCs cultured in a pair-wise comparison of lab-based and GMP conditions. Both 610 cell batches exhibited propagation but 609 and 611 showed gradual growth arrest or senescence, which is commonly observed for primary cells under *in vitro* culture.

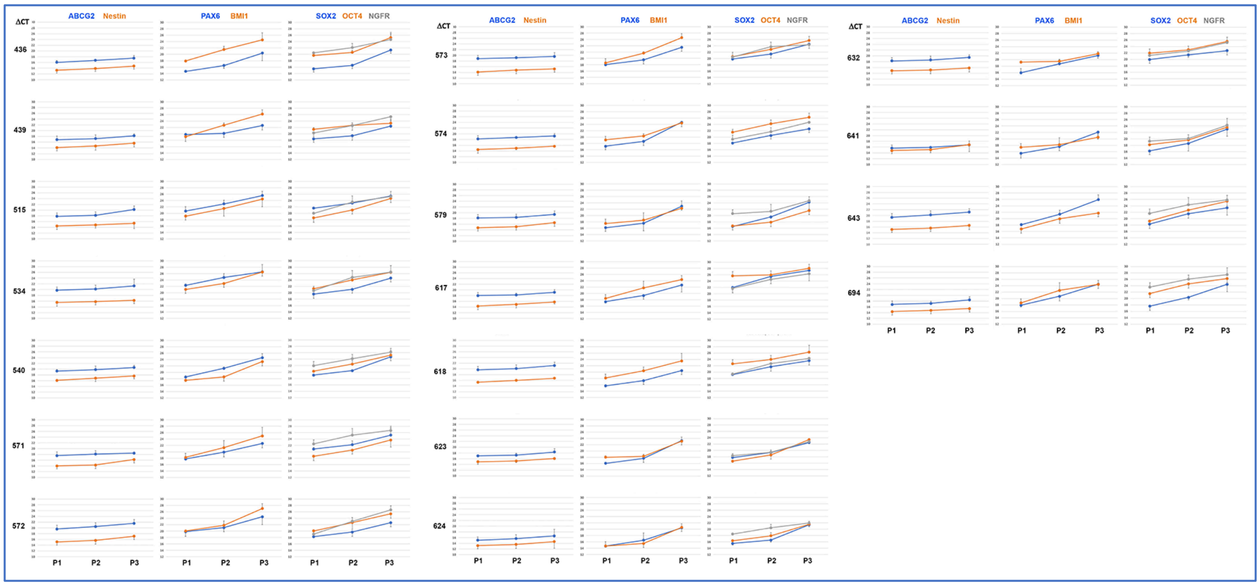

Supplemental Figure S5. Passage-dependent changes of stemness genes. The expression of ABCG2 and nestin remained consistent in P1, P2 and P3 CSSCs whereas Pax6, Bmi1, Sox2, Oct4 and NGFR were downregulated in P3, when compared to P1 cells.

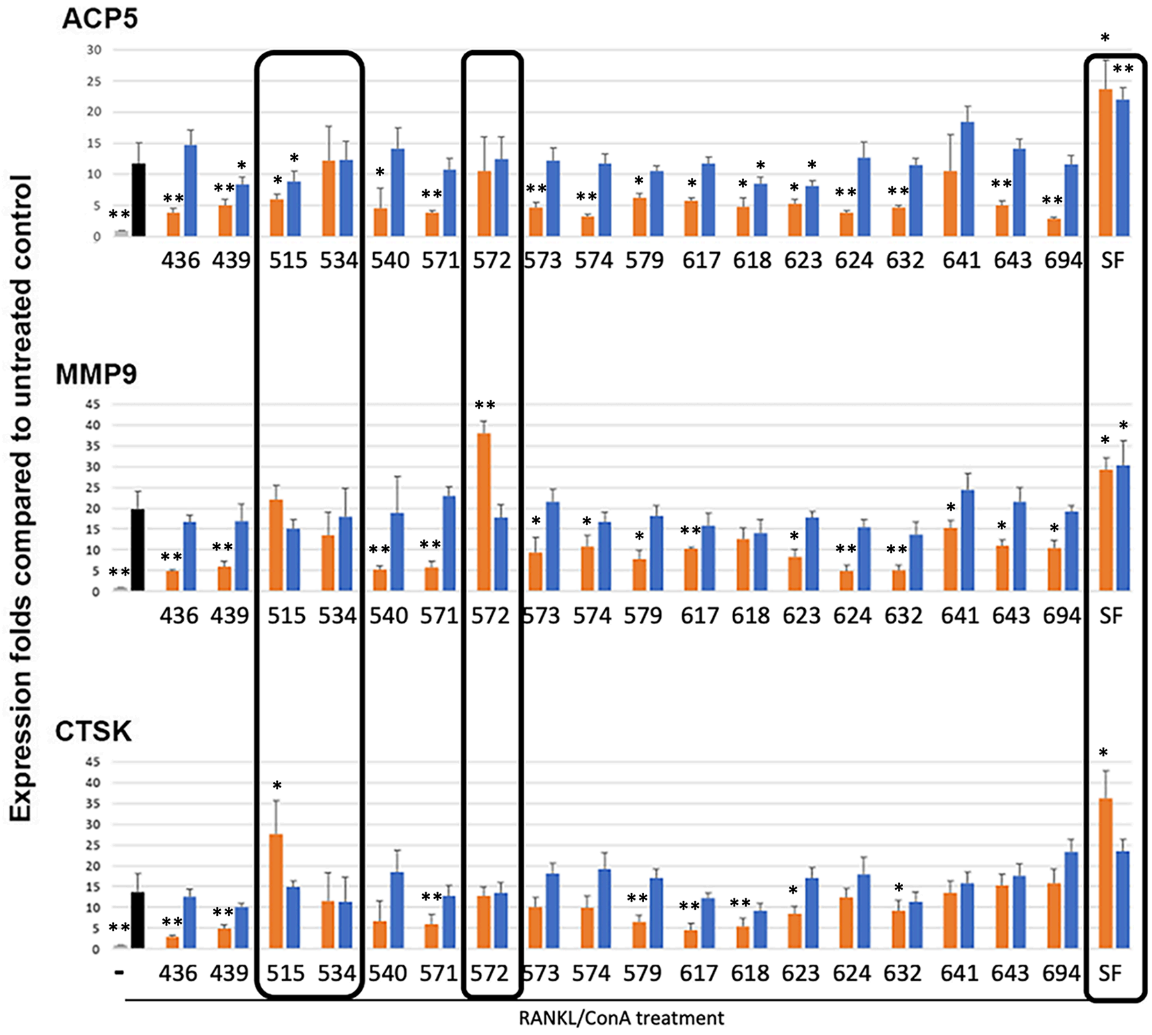

Supplemental Figure S6. Anti-inflammatory property of donor CSSC batches (n=18) at P2. Mouse RAW264.7 cells pre-incubated with heat-denatured CMconc showed upregulated osteoclast gene expression (ACP5, MMP9, and CTSK) (blue coloured) after RANKL/ConA induction, similar to the controls (black). All 3 genes were downregulated after treatments using native CM (orange) from most CSSC batches, but not with CM from HC515, 534, and 572 (in bracket). The treatment with CM from human stromal fibroblasts (SF) serves as a negative control without anti-inflammatory effect. Grey bars: naïve RAW cells; dark bars: RAW cells treated by RANKL/ConA (without CM). * P<0.05 and ** P<0.01 compared with RANKL/ConA-treated cells (Mann-Whitney U test).

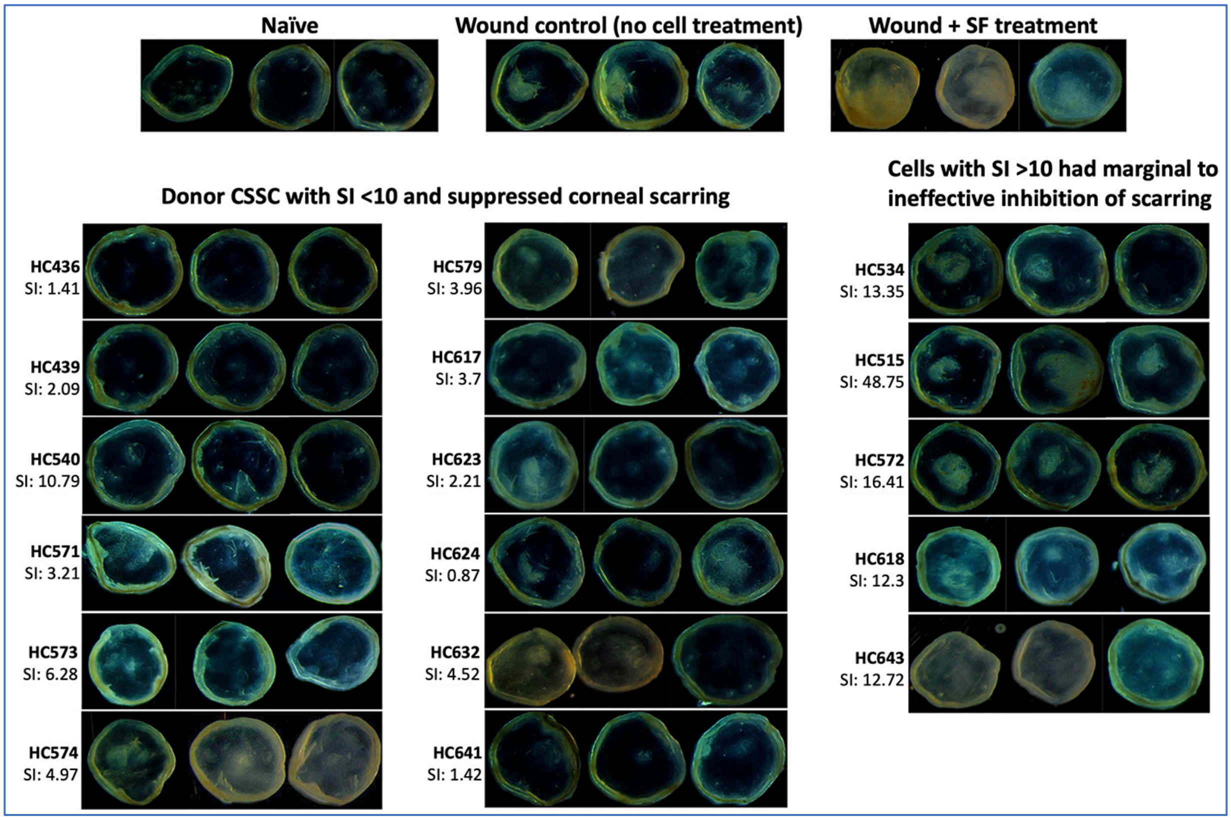

Supplemental Figure S7. Mouse corneal images showing the treatment outcome with different donor CSSC having their respective SI values, compared with naïve and untreated wound controls. The treatment with donor stromal fibroblasts serves as the negative control of scar inhibition. Donor CSSC with SI <10 resulted in scar inhibition, whereas cells with higher SI values (SI >10) showed moderate to ineffective scar prevention. Corneas treated with HC515 and HC572 showed similar scar manifestation as the wound control.

Supplemental Table S1. Donor cornea information

Lab no.	Age	Gender	Cause of Death	Days in Optisol preservation
**436**	33	M	Multiple cerebral infarcts	7
**439**	57	F	Anoxia brain injury	9
**466**	51	F	Myocardial infarct	8
**515**	67	M	Atherosclerotic cardiovascular disease	6
**534**	25	M	Head trauma	9
**540**	21	M	Dilated Cardiomyopathy	8
**571**	63	M	Anoxic brain injury	12
**572**	21	M	Anoxic brain injury	10
**573**	68	M	STEMI	10
**574**	33	M	Asphyxiation	9
**579**	29	F	Drug intoxication	10
**618**	57	M	Acute myocardial infarct	8
**624**	65	M	Hypoxic ischemic encephalopathy	10
**641**	45	M	Gun shot	12
**643**	37	M	Blunt force trauma of head	8
**694**	10	F	Hypoxic brain injury	6
**GMP11**	18	M	Blunt force trauma of head	7
**GMP13**	34	F	Pulmonary embolism	6
**GMP14**	61	M	Acute myocardial infarct	7
**GMP19**	63	M	Pulmonary embolism	8
**GMP23**	30	M	Blunt force trauma	7

The central stroma from sample 579, 618, 624, 641, and 643 were used for the isolation of stromal keratocytes and cultivation.

Supplemental Table S2. qPCR primers and antibodies used in this study

(A) Expression primers

	GenBank #	Forward (5'-3')	Reverse (5'-3')
Mouse markers			
ACP5	NM_013556.2	GTTGGATACAGGCCAGACTTTGTTG	GATTCAACTTGCGCTCATCTTAGGC
aSMA	NM_009696.3	TGTGCTGGACTCTGGAGATG	GAAGGAATAGCCACGCTCAG
Col3A1	NM_009930.2	CGTAAGCACTGGTGGACAGA	CGGCTGGAAAGAAGTCTGAG
CTSK	X94444.1	ATGTGGGGGCTCAAGGTTCTG	CATATGGGAAAGCATCTTCAGAGTC
MMP9	NM_013599.5	CTTCTGGCGTGTGAGTTTCCA	ACTGCACGGTTGAAGCAAAGA
SPARC	XM_030245730.1	ATGAGGGCCTGGATCTTCTT	CACGGTTTCCTCCTCCACTA
Human markers			
ABCG2	NM_001257386.2	TGCAACATGTACTGGCGAAGA	TCTTCCACAAGCCCCAGG
ALDH3A1	NM_001135168.1	CATTGGCACCTGGAACTACC	GGCTTGAGGACCACTGAGTT
B3GNT7	NM_145236.2	AGTCTCACCCCTGGTCAGTT	AGCAGTTAGTGGTGGTCACG
BMI1	NM_005180.8	TCATCCTTCTGCTGATGCTG	GCATCACAGTCATTGCTGC
CHST6	NM_021615.4	TACCGGCCTGTGTACTCTGA	ACTAATTTCGGGGGTGCGAG
EGFR	NM_201282.2	AACACCCTGGTCTGGAAGTACG	TCGTTGGACAGCCTTCAAGACC
KERA	NM_007035.3	ATCTGCAGCACCTTCACCTT	CATTGGAATTGGTGGTTTGA
LUM	NM_002345.3	CCTGGTTGAGCTGGATCTGT	TGGTTTCTGAGATGCGATTG
Nestin	NM_006617	AAGATGTCCCTCAGCCTGG	GAGGGAAGTCTTGGAGCCAC
NGFR	NM_002507.3	CCTACGGCTACTACCAGGATG	CACACGGTGTTCTGCTTGTC
PAX6	NM_000280.6	GTTGGTATCCCGGGGACTTC	TCCGTTGGAACTGATGGAGT
SOX2	NM_003106.4	AACCCCAAGATGCACAACTC	GCTTAGCCTCGTCGATGAAC

(B) Antibodies (anti-human)

Antibody [clone]	Source	Applications in this study
Aquaporin A (AQP1)	Santa Cruz Biotech sc-32738	Immunofluorescence
Keratocan (Kera)	Sigma HPA039321	Immunofluorescence

Supplemental Table S3. Calculation of *in vitro* scarring indices (SI) of CSSC and comparison to the respective *in vivo* percentages of scarring area after cell treatment to mouse corneas. Two QC parameters - ΔCT of ABCG2 and nestin (mean ± SD, normalized with the housekeeping 18s) of donor CSSC, and rate of inflammation (expression fold changes of ACP5, MMP9, and CTSK after treatment of native versus denatured CSSC-derived conditioned media concentrates in RAW 267.4 cells. Scarring index was calculated as the sum of $2^{\Delta CT(ABCG2)}$+$2^{\Delta CT(NES)}$/100,000 + $2^{\Sigma RInflam}$/10.

CSSC at P2	ΔCT (ABCG2)	ΔCT (NES)	$2^{\Delta CT(ABCG2)}$ +$2^{\Delta CT(NES)}$ /100,000	ACP5	MMP9	CTSK	$2^{\Sigma RInflam}$/10	*In vitro* Scarring indices	*In vivo* % Scarring
**436**	16.72 ±0.28	13.9 ±0.35	1.232	0.26	0.30	0.24	0.174	1.406	19.9±16.6
**439**	17.21 ±0.32	14.65 ±0.41	1.773	0.60	0.61	0.43	0.312	2.085	4.2±1.3
**515**	18.17 ±0.21	14.83 ±0.07	3.241	0.78	3.44	4.61	45.509	48.750	119.9±55.5
**534**	20.21 ±0.33	15.86 ±0.15	12.724	1.00	0.64	1.01	0.628	13.352	65.5±52.8
**540**	19.83 ±0.28	16.86 ±0.14	10.510	0.42	0.59	0.46	0.277	10.787	14.9±6.4
**571**	18.09 ±0.17	14.32 ±0.29	2.995	0.36	0.25	0.46	0.210	3.205	40.5±39.2
**572**	20.47 ±0.31	15.66 ±0.34	15.042	0.77	2.07	0.93	1.364	16.406	102.3±14.1
**573**	19.13 ±0.34	14.8 ±0.11	6.023	0.38	0.44	0.56	0.260	6.283	8.1±9.2
**574**	18.74 ±0.26	14.96 ±0.06	4.697	0.28	0.65	0.52	0.273	4.970	28±19.2
**579**	18.36 ±0.15	15.08 ±0.15	3.711	0.59	0.43	0.38	0.264	3.975	45.5±28.8
**617**	18.24 ±0.26	14.93 ±0.23	3.408	0.49	0.65	0.38	0.287	3.695	53.2±18.5
**618**	20.1 ±0.34	15.99 ±0.11	11.889	0.56	0.90	0.59	0.414	12.303	33.1±17.1
**623**	17.22 ±0.2	15.19 ±0.15	1.900	0.65	0.47	0.5	0.307	2.207	50.7±51.9
**624**	15.61	13.59	0.623	0.30	0.32	0.69	0.248	0.871	45.9±34.6

	±0.39	±0.21										
**632**	18.56 ±0.35	15.11 ±0.29	4.218		0.41	0.37	0.8	0.299		4.517		51.4±44.9
**641**	15.93 ±0.26	15.12 ±0.09	0.980		0.66	0.65	0.81	0.435		1.415		17.2±9.4
**643**	20.18 ±0.49	15.63 ±0.2	12.386		0.35	0.51	0.87	0.332		12.718		28.3±31.2
**694**	17.21 ±0.23	14.79 ±0.17	1.799		0.25	0.54	0.68	0.277		2.076		27.6±8.7
**HCF**					1.12	0.98	1.93	1.634				

Note: A comparison to HCF (human corneal stromal fibroblasts) with expression fold changes of ACP5, MMP9, and CTSK in the osteoclastogenesis assay. The result showed that HCF was non-anti-inflammatory compared with most CSSC batches.
